# Evaluating the Scale of Natural and Mechanical Airflows for Surface-Based Atmospheric Pollutant Removal

**DOI:** 10.1021/acs.est.5c16697

**Published:** 2026-07-24

**Authors:** Samuel D. Tomlinson, Aliki M. Tsopelakou, Tzia M. Onn, Steven R. H. Barrett, Adam M. Boies, Shaun D. Fitzgerald

**Affiliations:** † Department of Engineering, 2152 University of Cambridge, Cambridge CB2 1PZ, U.K.; ‡ Centre for Climate Repair, Cambridge CB3 0WA, U.K.; ¶ School of Computing and Mathematical Sciences, University of Greenwich, London SE10 9LS, U.K.; § Department of Mechanical Engineering, 6429 Stanford University, Stanford, California 94305, United States

**Keywords:** pollutant removal, cities, HVAC systems, transport, sorption, catalysis, filtration

## Abstract

Removal strategies for atmospheric pollutants are increasingly being considered to mitigate global warming and improve public health. However, the global potential of surface-based removal techniques has not yet been quantified under atmospheric transport. We evaluate the atmospheric pollutant transport to surfaces and assess the potential of surface-based removal technologies across infrastructure. Cities provide the highest transport-limited removal potential, with median annual atmospheric flow rates of 30 GtCO_2_, 0.06 GtCH_4_, 0.007 GtNO_*x*_, and 0.0001 GtPM_2.5_ to their total surface area. Cities and HVAC systems have flow rates large enough to potentially remove more than 1 GtCO_2_/y (1 GtCO_2_e/y for CH_4_, 20-year GWP), if laboratory-scale removal efficiencies are achieved under current atmospheric concentrations. HVAC filters have the potential to achieve costs as low as $600 per tCO_2_ removed ($2000 per tCO_2_e) if CO_2_-sorption (CH_4_-catalyst) technologies are incorporated into their surfaces and maintained through replacement, compared with $3000 per tCO_2_ ($10,000 per tCO_2_e) for cities, using literature material and application costs. These estimates exclude regeneration energy, associated electricity use, and downstream processing. These findings suggest that integrating surface-based pollutant removal technologies into infrastructure could support climate mitigation, although further work is needed to assess feasibility, deployment, and cost in application.

## Graphical abstract

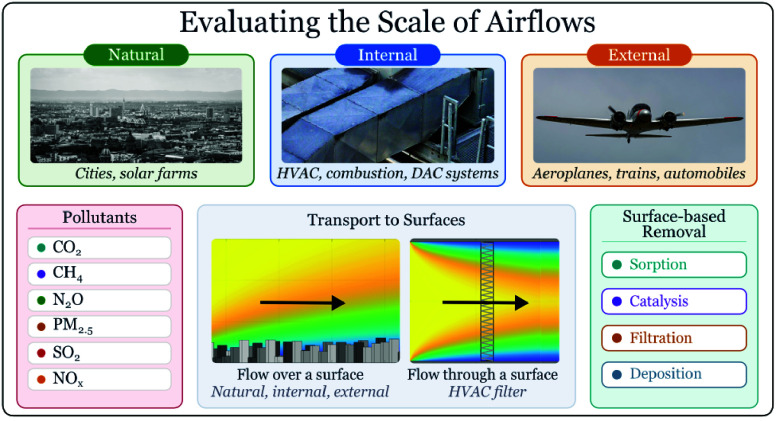

## Introduction

Greenhouse gases (GHGs) such as carbon dioxide (CO_2_), methane (CH_4_), and nitrous oxide (N_2_O) continue to rise at substantial rates, presenting sustainability and health challenges by accelerating climate change.^1,2^ In 2023, CO_2_ levels surpassed 420 ppm, driven by fossil fuel use and deforestation, contributing to biodiversity loss and environmental degradation.^2,3^ Although CH_4_ and N_2_O are emitted in smaller quantities than CO_2_, their 20-year global warming potentials (GWPs) are 84 and 273 times greater than CO_2_, highlighting their disproportionate climate impacts despite lower atmospheric concentrations.^2,4^ CO_2_ is emitted from anthropogenic activities (e.g., fossil fuel combustion, land-use change) and natural processes (e.g., respiration, decomposition), and CH_4_ and N_2_O arise from a combination of anthropogenic sources, such as agriculture and fossil fuel processing, and natural microbial processes.^5–7^ Many GHG sources are spatially distributed and occur at low concentrations, making them difficult to remove.

In addition to GHGs, atmospheric pollutants such as particulate matter (PM), sulfur dioxide (SO_2_), nitrogen oxides (NO_*x*_), and ozone (O_3_) contribute to a range of environmental and health risks.^8,9^ PM, particularly particles smaller than 2.5 μm (PM_2.5_), is a leading cause of respiratory and cardiovascular diseases, responsible for millions of premature deaths each year.^9–11^ PM is emitted from anthropogenic sources, such as fossil fuel combustion and processing, and natural sources, like wildfires. It can also form secondarily from precursor gases including SO_2_ and NO_*x*_, which have similar sources to primary PM and contribute to acid rain and ground-level O_3_.^12–14^ O_3_, a secondary pollutant formed photochemically from NO_*x*_ and volatile organic compounds (VOCs), exacerbates respiratory illness and contributes indirectly to radiative forcing, though its atmospheric lifetime is shorter than those of long-lived GHGs.^15^

Given the diffuse nature of anthropogenic, biogenic, and natural sources, coupled with the insufficient progress in emissions reductions,^2,3,5,6,9,11–13^ there is growing interest in atmospheric pollutant removal. Atmospheric pollutant removal strategies can broadly be grouped into (i) surface-based approaches,^16–23^ which capture or transform pollutants at solid interfaces, and (ii) airborne methods,^24,25^ such as radical generation or advanced photochemistry. While airborne methods show promise, this study focuses on surface-based removal methods such as sorption, catalysis, and filtration, given that these approaches are more widely explored and developed. Despite the interest in surface-based atmospheric pollutant removal,^16–23^ the scale of potential removal remains unexplored. This study quantifies the transport of CO_2_, CH_4_, NO_*x*_, and PM_2.5_ to different surfaces to estimate upper-bound removal potentials, providing a comparative framework across environments rather than prescribing deployment pathways.

Sorption technologies include liquid solvent-based systems (e.g., aqueous amines or hydroxide solutions) and solid sorbent-based systems (e.g., amine-functionalized materials), which capture CO_2_ through chemical absorption or adsorption, as used, e.g., in direct air capture (DAC).^16,26^ For DAC, the removal efficiency is defined as the fraction of CO_2_ removed from the airflow during passage through the capture device. Reported values can range from 10^–3^ to 10^0^ depending on the capture material, reactor configuration, and operating conditions.^27^ However, this process can require substantial energy and involve high operational costs.^28,29^ Sorption-based systems require periodic regeneration, and the captured CO_2_ must subsequently be concentrated, compressed, transported, and sequestered. These steps impose additional energy and cost constraints, affecting the feasibility of durable atmospheric CO_2_ removal.^28,29^ Membrane-based systems, which separate CO_2_ through selectively permeable barriers, may offer greater energy efficiency than DAC, but face challenges related to initial costs and scalability.^17^ In contrast, leveraging pre-existing natural and mechanical airflows, where the associated energy demands are already accounted for, may enable scalable and cost-effective surface-based methods for atmospheric pollutant removal.

Catalytic oxidation can convert CH_4_ into CO_2_, reducing radiative forcing rather than achieving net carbon removal, as CH_4_ has a 20-year GWP approximately 84 times higher than CO_2_;^4^ however, performance optimization across environments remains challenging. Reported CH_4_ removal efficiencies for catalysts can vary widely, from 10^–11^ to 10^0^, reflecting differences across catalysts and experimental conditions.^30,31^ Beyond catalyst materials and application, the feasibility of atmospheric pollutant removal technologies depends on fabrication, installation, and energy inputs. Catalytic CH_4_ oxidation may also require thermal, photo, or electrochemical activation and additional infrastructure to enable effective air–surface interaction. Recent cost analyses suggest that using photocatalytic oxidation for atmospheric CH_4_ removal is prohibitively expensive due to the high flow rates required for effective removal.^25,31,32^ Targeting higher-concentration CH_4_ sources can yield meaningful climate benefits at source concentrations above 10 ppm, with cost-effective operation using current technologies requiring concentrations above 10^3^ ppm.^33^ Hydroxyl and chlorine radicals can oxidize CH_4_ in the gas phase, and laboratory-scale photochemical systems have reported relatively low energy inputs for low-concentration pollutant removal.^31,34^ Alternatively, atmospheric radicals have been suggested to break down CH_4_ but at slow, condition-dependent rates^24,25^ and methanotrophic bacteria can remove CH_4_, albeit under controlled conditions.^19^ This study examines catalytic oxidation as a strategy for atmospheric pollutant removal, exploring applications with varying surface fluxes and areas for scalable implementation.

High-efficiency particulate air (HEPA) filters are widely used for PM capture, though their performance depends on particle size distributions, pressure-drop constraints, and replacement cycles;^20^ additional coatings could extend their removal potential to some gaseous pollutants. Other methods for removing PM include electrostatic precipitators and adsorption materials such as activated carbon.^21^ These technologies can offer removal efficiencies of 10^–1^ to 10^0^,^35^ but require periodic regeneration or replacement due to saturation.^36^ PM is also removed from the atmosphere through dry (e.g., gravitational settling, surface absorption) and wet deposition (e.g., scavenging by rain), with removal rates varying by region and environmental conditions.^37–39^ While these mechanisms for PM removal are well-established, this study estimates how much PM_2.5_ is removed from the atmosphere by filtration technologies, comparing these with the potential removal rates of other pollutants.

Similar to CH_4_, N_2_O can be removed via catalytic reduction, which converts N_2_O into nitrogen (N_2_) and oxygen (O_2_).^25^ Photochemical destruction approaches have also been investigated for N_2_O and fluorinated anesthetic gases in controlled exhaust streams.^40^ Another surface-based removal process is biological denitrification, where bacteria reduce N_2_O to N_2_ under anaerobic conditions.^41^ SO_2_, NO_*x*_, and O_3_ can be transformed through catalytic processes (e.g., selective catalytic reduction for NO_*x*_, oxidation pathways for SO_2_, photocatalysis for O_3_), with variability in efficiency, byproducts, and technological maturity.^22^ Another removal method is scrubbing, where SO_2_ and NO_*x*_ are removed from gas streams by passing them through chemically reactive liquid solutions.^23^

In summary, although many surface-based pollutant removal methods exist (or are being proposed), they can be inefficient, energy-intensive, and/or costly. Furthermore, the literature lacks an assessment of the potential scale of reductions achievable with surface-based atmospheric pollutant removal, despite the need for scalable solutions. In transport-limited systems, removal is constrained by the atmospheric delivery of pollutants to the surface. Removal can also be limited by reaction kinetics, catalyst activity, regeneration frequency, or application-specific parameters such as residence time, surface area, and pressure drop. Here, we use the transport flux, surface area, and laboratory removal efficiencies to estimate the potential removal, providing an indicative estimate used to compare different potential applications.

In this study, we examine approaches that could utilize the substantial volumes of air transported in urban environments, heating, ventilation, and air conditioning (HVAC) systems, and transportation networks. Rather than focusing on the performance of specific surface-based technologies that remove specific pollutants, this study evaluates the scalability of atmospheric pollutant removal via surface-based interactions through transport limitations. By applying representative laboratory-scale removal efficiencies from the literature, we estimate potential removal rates for sorption, catalysis, and filtration across a range of environmental and flow conditions. Throughout this study, global-scale removal potentials are calculated assuming complete use of existing surfaces under current atmospheric concentrations, representing transport-limited upper bounds rather than realistic deployment scenarios. We compare the potential pollutant removal rates with intergovernmental targets and existing removal mechanisms, evaluating the scalability of these surface-based technologies. We identify configurations where material and application costs alone may constrain large-scale deployment, recognizing that full techno-economic feasibility depends on energy use and system integration.

## Methods

The methods outlined in this section are used to calculate the pollutant flux or flow rate to the surfaces of different natural and mechanical environments. These fluxes or flow rates are independent of the removal technology and indicate the rate at which a given atmospheric pollutant could be delivered to the surface of a given environment to remove it. In this work, we distinguish between flow-over and flow-through transport regimes. For city surfaces, pollutant delivery is governed by boundary layer transport, where flux to the surface depends on turbulent mixing and concentration gradients. In contrast, HVAC filtration operates as a flow-through configuration in which air is driven through a porous medium and pollutant transport is governed by the velocity component normal to the medium and the concentration. External engineered systems such as aircraft represent a further regime in which free-stream velocities generate thin turbulent boundary layers. For gaseous species, transport to surfaces is governed primarily by advection and molecular or turbulent diffusion. For PM, transport may additionally involve gravitational settling, Brownian diffusion, inertial impaction, and interception.^42^ Pollutant-specific capture processes are incorporated separately where appropriate (e.g., through the diffusivity *D* in eq 2 or the HVAC filtration efficiency *E* in eq 12).

### Scaling Theory

To characterize atmospheric pollutant transport dynamics across various systems, we define velocity (*u*, m/s), length (*l*, m), width (*w*, m), and surface area (*s*, m^2^) scales specific to each flow application (e.g., cities, HVAC systems, transport). We also estimate the number of occurrences (*n*) for each flow type to enable scaling for global estimations. The limitations of this scaling approach and additional context are provided in Section S3. The thickness of the turbulent (*Re* > 10^5^) boundary layer (δ, m) is evaluated using δ = 0.4*l*/*Re* ^1/5^, where the Reynolds number *Re* = *ul*/ν and ν is the kinematic viscosity (m^2^/s).^43^ This empirical relation describes a turbulent boundary layer developing over a smooth flat plate under zero pressure gradient. In this work, it is used as a scaling for surface transport across environments, rather than as an exact representation of transport in urban flows, which are influenced by surface roughness and can be more appropriately described using parameters such as roughness length and displacement height.^43^

The normal pollutant flux *j*_*y*_ (mol/(m^2^s)) at the surface is determined by the diffusive flux, −*Dc*_*y*_, as the normal velocity at the surface is zero. This normal pollutant flux depends on the diffusivity *D* (m^2^/s), concentration *c* (mol/m^3^), and the boundary layer thickness δ_c_ = δ/*Sc* ^1/3^ (m),^44^ where the Schmidt number *Sc* = ν/*D*. Molecular diffusivities of gases in air are typically *O*(10^–5^) m^2^/s, however, the Brownian diffusivity of particles decreases with particle diameter according to the Stokes–Einstein relation, with values around *O*(10^–9^) m^2^/s for 0.1 μm particles and *O*(10^–11^) m^2^/s for 1 μm particles.^42^ Particle diffusivities are therefore several orders of magnitude smaller than gaseous diffusivities. An effective diffusivity *D*_e_ (m^2^/s) is included to account for enhanced turbulent mixing, which alters pollutant transport rates.^45^ We will assume well-mixed concentrations and constant diffusivities throughout.

Using these scales, the streamwise flow rate of pollutants *Q* (m^3^/s) through the boundary layer of each application is given by

1 Q≈nuδw

The normal flow rate of pollutants *q*_*y*_ (mol/s) to the surface of each application is given by

2 $$q_{y}\approx \frac{ns\left(D+D_{e}\right)c}{\delta /{Sc}^{1/3}}$$

For some applications (e.g., cities, solar farms), multiple characteristic length scales are applied (e.g., buildings, solar panels), whereas for others (e.g., ducts, aeroplanes), only a single length scale is used. For applications with a single length scale, eqs 1–2 are evaluated using parameter distributions from Tables S1–S4. For multiple length scale applications, eqs 1–2 are evaluated separately for each length scale and the results are combined.

### Empirical Relationships

Empirical models can be used to link flow characteristics with the potential for pollutant removal.^44^ These relationships have been applied in CH_4_ and N_2_O removal studies via photocatalysis.^25,30^ These relationships are evaluated using *l*, *s*, and *u*, supplemented with measured values such as wall shear stresses (τ_w_ in kg/(ms^2^)), mass transfer coefficients (*m*_c_ in m/s), and heat transfer coefficients (*h*_c_ in W/(m^2^K)). A detailed discussion of these empirical relationships and their applications can be found in Section S3. Analogies exist between dimensionless numbers: the Nusselt number (*Nu* = *h*_c_ *l*/*k*), the Sherwood number (*Sh* = *m*_c_ *l*/*D*), and the drag coefficient (*C*_d_ = τ_w_/(ρ*u* ^2^)), where *k* is the thermal conductivity (W/(mK)) and ρ is the fluid density (kg/m^3^).^44^ These analogies allow for the formulation of the normal pollutant flow rate to the surface of each application as follows

3 $$q_{y}\approx 0.03{Re}^{4/5}{Sc}^{1/3}nsDc/l$$

Following Tsopelakou et al.,^30^ we can use eq 3 to estimate the streamwise flow rate through each application as

4 $$Q\approx uA\approx lm_{c}P$$

where *P* is the wetted perimeter and *A* is the cross-sectional area of the channel or boundary layer. Equations 3–4 are evaluated using the parameter distributions listed in Tables S1–S4.

### Energy, Power, and Drag Measurements

We now outline formulas to determine the flow rate based on energy, power, and drag measurements. More details are given in Section S3. For external flows over urban areas or solar farms, we establish a control volume with a length *l*, width *w*, and height δ. In the case of unidirectional, steady-state flow over a surface with a heat flux ϕ (W/m^2^), energy conservation dictates that the energy entering the control volume must balance with the energy exiting it. This relationship can be expressed as

5 $$Q\approx \frac{ns\phi }{\rho c_{p}\Delta T},q_{y}\;\approx \;\frac{Ql\left(D+D_{e}\right)c}{\delta^{2}u}$$

where *c*_p_ (m^2^/(s^2^ K)) is the specific heat capacity of air at constant pressure and Δ*T* (K) is the temperature difference between the surface and the surrounding atmosphere. The flow rates are related using the scaling theory discussed in Section S3, such that *Ql*(*D* + *D*_e_)*c*/(δ^2^ *u*) ≈ *Qs*(*D* + *D*_e_)*c*/(δ^2^ *uw*) ≈ *s*(*D* + *D*_e_)*c*/δ ≈ *q*_*y*_.

In the context of internal flow through HVAC systems, the total power consumed by the fans, denoted as *P* (kW), and the specific fan power (SFP) (W/(m^3^/s)), can be utilized to evaluate the streamwise flow rate through the duct as follows

6 $$Q\approx \frac{LP}{\mathrm{SFP}}$$

where *L* is the leakage factor.^46^ For external airflows acting on transportation systems, the streamwise flow rate through the boundary layer can be estimated using the drag force acting on the aeroplane, train or automobile.^47^ This is expressed by the equation

7 $$Q\approx \frac{2nF_{d}}{\rho uC_{d}}$$

where *F*_d_ (N) is the drag force. Equations 5–7 are evaluated using the parameter distributions listed in Tables S1–S4.

### Industry Standards

For internal airflows through HVAC systems, the streamwise flow rate can be evaluated using three estimates. First, *Q* can be expressed in terms of the cubic feet per minute per person (CFM_pp_, ft^3^/min), given by

8 $$Q\approx {\mathrm{CFM}}_{\mathrm{pp}}n_{\mathrm{pp}}$$

where *n*_pp_ (−) is the total number of occupants in the building. Second, *Q* can be calculated based on the CFM per unit area (CFM_pm^2^_, ft^3^/min/m^2^), described by the equation

9 $$Q\approx {\mathrm{CFM}}_{{\mathrm{pm}}^{2}}a$$

where *a* (m^2^) is the total floor area of the building. Finally, *Q* can be assessed using the air changes per hour (ACH, 1/h), given by

10 Q≈ACHv

where *v* (m^3^) is the volume of air within the building. Equations 8–10 are evaluated using the parameter distributions listed in Tables S1–S4.

### Fully Developed Profiles

For internal airflows within HVAC systems, we approximate solutions of the Navier–Stokes equations and boundary conditions. The normal flow rate of pollutants to the channel walls is given by

11 $$q_{y}\approx \frac{ns\left(D+D_{e}\right)c}{h}$$

where 2*h* is the channel diameter. For internal airflows through HVAC filters, we adjust the velocity from the duct flow (with velocity *u*, diameter 2*h,* cross-channel area *A*) to the pleat flow (with velocity *u*_**n**_, diameter 2*h*_p_, filter sheet area *A*_p_, number *m*). We apply the principle of mass conservation to match average velocities, leading to *Au* ≈ *mA*_p_ *u*_**n**_. The flow rate of pollutants to the fiber sheet is

12 $$q_{n}\approx nmEA_{p}u_{n}c$$

where *E* is the collection efficiency, related to the permeability and porosity of the filter medium.^42^ Depending on particle size and flow conditions, dominant mechanisms include Brownian diffusion, interception and inertial impaction. These mechanisms are incorporated into the collection efficiency term *E* in eq 12. For μm-scale particles typical of atmospheric PM_2.5_, filtration is often inertia- or interception-dominated, such that the pollutant removal rate depends primarily on the flow-through velocity rather than molecular diffusivity. Equations 11–12 are evaluated using the parameter distributions listed in Tables S1–S4, and the resulting distribution is then combined with the other methods from this section.

### Box Model

To evaluate how surface-based pollutant removal could alter atmospheric concentrations, we consider a well-mixed atmospheric box model. We assume the atmospheric concentration *C* = *C*(*t*) evolves according to

13 $$\frac{dC}{dt}=\frac{E}{M_{atm}}-\left(k_{nat}+k_{surf}\right)C$$

where *E* = *E*(*t*) is the emission rate, *M*_atm_ is the total atmospheric burden of the pollutant, *k*_nat_ is the first-order rate constant for natural removal processes, and *k*_surf_ is the first-order rate constant for potential surface-based removal. Assuming constant emissions, the solution is given by

14 $$C=C_{eq}+\left(C_{0}-C_{eq}\right)\exp\left(-\left(k_{nat}+k_{surf}\right)t\right)$$

where *C*_eq_ = *E*/(*M*_atm_(*k*_nat_ + *k*_surf_)) is the equilibrium concentration and *C*(0) = *C*_0_. For CO_2_, the present atmospheric burden is approximately 3200 GtCO_2_, with anthropogenic emissions of approximately 40 GtCO_2_/y.^48^ The lifetime of atmospheric CO_2_ is on the order of 50 to 100 years, giving a natural removal rate constant *k*_nat_ ≈ 0.01 to 0.02 y^–1^. For CH_4_, the present atmospheric burden is approximately 5.4 GtCH_4_ and the atmospheric lifetime is approximately 9 to 12 years,^49^ giving a natural removal rate constant *k*_nat_ ≈ 0.1 y^–1^. The potential surface-based removal rate constant is estimated as *k*_surf_ = *R*_surf_/*M*_atm_, where *R*_surf_ is the potential removal rate for the pollutant removal technology. Tropospheric mixing times are approximately one year globally and weeks to months regionally, supporting the use of a well-mixed approximation as a scaling analysis for CO_2_ and CH_4_; more detailed spatial predictions would require chemical transport modeling and are beyond the scope of this work.

### Parameter Estimation and Uncertainty

To account for variability in atmospheric pollutant transport, we estimated ranges for parameters across natural, internal, and external environments, including characteristic length, surface area, velocity, and total and produced numbers for each environment (Tables S2–S4). Minimum, mean, and maximum values were compiled from published literature and, where available, measurement data sets (e.g., wind speeds in cities,^50^ Figure 1E). For parameters with limited data, we adopted a representative value from the literature for the mean and estimated a plausible range, typically spanning approximately 1 order of magnitude, to reflect environmental variability and reporting uncertainty. Each parameter was represented as a triangular distribution (minimum–mean–maximum) to capture uncertainty, and this distribution was propagated through the atmospheric pollutant surface flux and flow rate (eqs 1–12). This generated a distribution of outcomes, which we illustrate in the Results using box-and-whisker plots, where the central line denotes the median, the box bounds represent the interquartile range (IQR), and the whiskers indicate the full range of simulated values. Example mean values are overlaid for each method to aid comparison.

**Figure 1:**
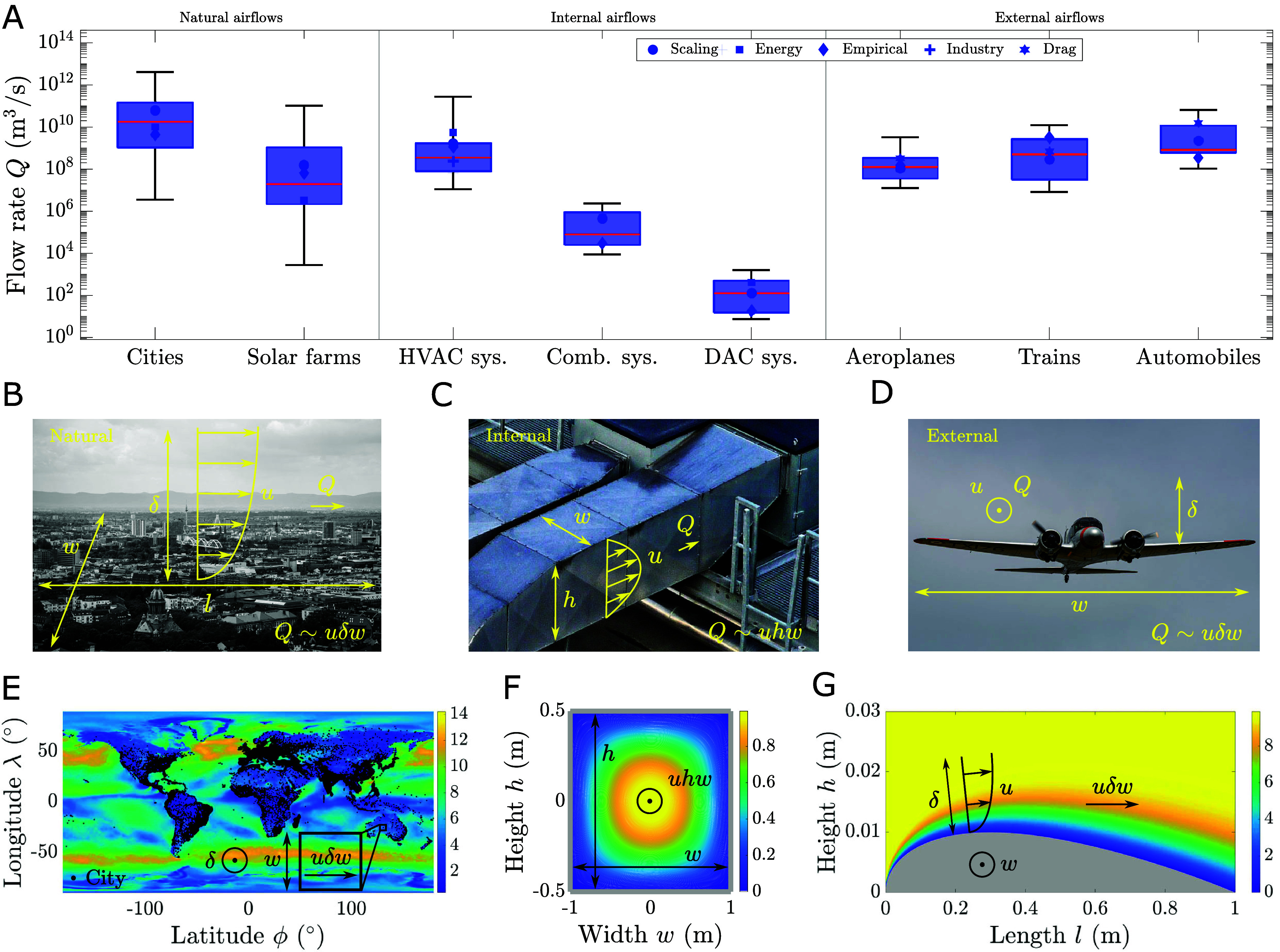
Flow rates through global environments. (A) Streamwise flow rates (*Q*) for natural airflows (e.g., cities, solar farms), internal airflows (e.g., HVAC, combustion, DAC systems), and external airflows (e.g., aeroplanes, trains, automobiles). Box-and-whisker plots show the distribution of flow rates, where boxes represent the interquartile range, central lines denote medians, whiskers show the full range of values, and symbols indicate mean flow rates derived from different estimation methods. Scaling (eqs 1–2), energy (eqs 5–6), empirical (eqs 3–4), industry (eqs 8–10), and drag (eq 7) approaches (using Tables S1–S4) are used to estimate *Q*. Schematics of the velocity field (*u*), length scales (*l*, δ, *w*, and *h*) and flow rate for a (B) city, (C) HVAC system, and (D) aeroplane. (E) Global wind speed distribution (m/s), with city data highlighted in black. (F) Streamwise velocity profile (m/s) through an HVAC system. (G) Boundary layer development (m/s) across an aerofoil surface.

## Results

Natural (e.g., cities, solar farms), internal (e.g., HVAC, combustion, DAC systems), and external environments (e.g., aeroplanes, trains, automobiles) exhibit substantial airflow volumes through their boundary layers, with global medians of 2 × 10^10^ m^3^/s (range: 3 × 10^6^ to 4 × 10^12^ m^3^/s) for natural, 4 × 10^8^ m^3^/s (1 × 10^7^ to 3 × 10^11^ m^3^/s) for internal, and 8 × 10^8^ m^3^/s (1 × 10^8^ to 7 × 10^10^ m^3^/s) for external environments (Figure 1A). Our analysis of variable parameters including length, surface area, velocity, and number of environments suggests that boundary-layer area and airflow velocity largely determine the flow rate through each environment: cities have the largest total surface area, whereas aircraft have higher velocities (Figure 1B–G; Tables S2–S4). The parameter distributions define the range of flow-rate values and indicate the relative potential of these environments for deployment of removal technologies.

### Surface Fluxes of Atmospheric Pollutants

#### Natural Flow over Surfaces

The following results describe the atmospheric pollutant fluxes to the surfaces of different environments; these values are determined solely by transport processes and are therefore independent of the removal technology. Figure 2A shows normalized atmospheric pollutant fluxes to the surfaces of natural environments (columns 1–2) in cities and solar farms. GHG fluxes exceed other pollutant fluxes due to higher diffusivities and atmospheric concentrations (Figure 2C). Shorter development lengths reduce boundary layer thickness, increase turbulence intensity, and enhance the transfer of atmospheric pollutants to surfaces.^45^ Cities exhibit average surface fluxes of 2 × 10^–8^ mol/(m^2^s) for CO_2_ (range: 4 × 10^–10^ to 1 × 10^–7^ mol/(m^2^s)), while solar farms exhibit average surface fluxes of 2 × 10^–7^ mol/(m^2^s) for CO_2_ (5 × 10^–9^ to 1 × 10^–6^ mol/(m^2^s)) (Figure 2C, Table S2). Although solar farms exhibit higher surface fluxes, the total surface area of cities will result in greater pollutant flow rates to their surfaces. Using empirical formulas detailed in the Methods, average atmospheric pollutant fluxes at the repeating unit scale (e.g., to the surface of individual buildings, 3 × 10^–2^ m^–1^) are larger than those at the combined unit scale (e.g., to the surface of entire cities, 7 × 10^–3^ m^–1^) due to steeper concentration gradients (Figure 2A,D,E). Urban turbulence and building spacing will also affect pollutant flow rates.^51^ In the Methods, these effects are incorporated via turbulent diffusivity and boundary-layer formulations at city and building scales.

**Figure 2:**
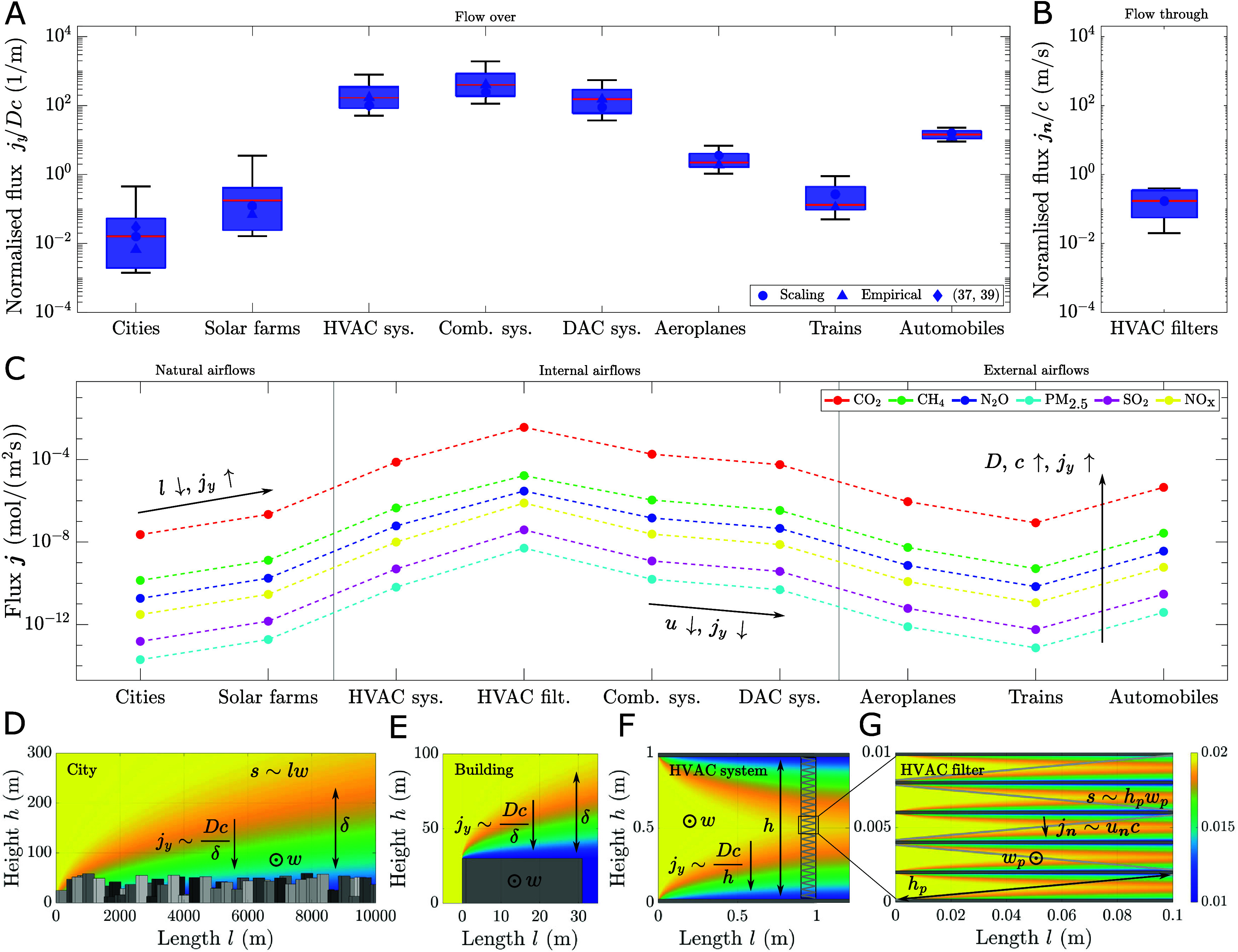
Atmospheric pollutant fluxes to the surfaces of global environments. (A) Atmospheric pollutant fluxes (***j***), normalized by diffusivity (*D*) and concentration (*c*), to the surfaces of natural airflows (e.g., cities, solar farms), internal airflows (e.g., HVAC, combustion, DAC systems), and external airflows (e.g., aeroplanes, trains, automobiles). (B) Atmospheric pollutant fluxes, normalized by concentration, to the surfaces of HVAC filters. (C) Atmospheric pollutant fluxes to surfaces, averaged across the different methods, for CO_2_, CH_4_, N_2_O, PM_2.5_, SO_2_, and NO_*x*_. Fluxes are estimated using scaling (eqs 1–2) and empirical (eqs 3–4) approaches, with parameter distributions from Tables S1–S4. Example concentration boundary layers, velocities (*u* and *u*_**n**_), lengths (*l*, δ, *w*, *h*, *w*_p_, *h*_p_), and normal pollutant fluxes to a (D) city surface (mol/m^3^), (E) building surface (mol/m^3^), (F) duct wall (mol/m^3^), and (G) HVAC filter sheet (mol/m^3^).

#### Internal Flow-Over and Flow-Through Surfaces

Figure 2A,B highlights the enhanced atmospheric pollutant transport to the surfaces of internal airflows (columns 3–5) in HVAC, combustion, DAC systems (Figure 2A), and HVAC filters (Figure 2B). Comparing HVAC, combustion, and DAC systems, which show average surface fluxes of 7 × 10^–5^, 2 × 10^–4^, and 6 × 10^–5^ mol/(m^2^s) for CO_2_ in Figure 2C, demonstrates that higher airflow velocities enhance atmospheric pollutant transport. The estimated value for DAC is comparable to 4 × 10^–5^ mol/(m^2^s) estimated in McQueen et al.^29^ Consistent with our definition of flow-over and flow-through systems (Methods), HVAC filters enhance encounter rates compared with duct walls. At the duct wall, the rate at which pollutants move perpendicular to the surface (normal flux) depends on diffusion and is proportional to *Dc*/*h* (e.g., an average of 7 × 10^–5^ mol/(m^2^s) for CO_2_, Figure 2C,F), where *D* is the diffusivity of the pollutant, *c* is its concentration, and *h* is the height of the channel. In contrast, for HVAC filters, the pollutant flux through the porous fiber sheet is proportional to *u*_**n**_ *c* (e.g., an average of 3 × 10^–3^ mol/(m^2^s) for CO_2_, Figure 2C,G), where *u*_**n**_ is the airflow velocity through the fiber sheet. Since *D* and *c* are typically small relative to *u*_**n**_ and *h*, *u*_**n**_ *c* is generally larger than *Dc*/*h*, highlighting the benefits of utilizing advective over diffusive transport to increase atmospheric pollutant fluxes to surfaces.

#### External Flow-Over Surfaces

Lastly, we evaluate the normalized atmospheric pollutant fluxes to the surfaces of external airflows over aeroplanes, trains, and automobiles (columns 6–8, Figure 2A). Automobiles demonstrate higher pollutant fluxes to surfaces than trains and aeroplanes (e.g., an average of 4 × 10^–6^, 8 × 10^–8^, 9 × 10^–7^ mol/(m^2^s) for CO_2_, Figure 2C), due to smaller characteristic lengths. However, aeroplanes generate more turbulent transport due to their higher velocities, resulting in a pollutant flux that is approximately 23% of that to automobile surfaces.

### Surface Flow Rates of Atmospheric Pollutants

#### Total Surfaces

For comparison of total flow to surfaces across applications, we include both flow-over and flow-through categories, even though their underlying transport mechanisms differ. Figure 3 quantifies the flow rates of atmospheric CO_2_, CH_4_, PM_2.5_, and NO_*x*_ to all global surfaces. These flow rates are evaluated by multiplying the fluxes from Figure 2 by the relevant surface area for each application (*s* in Figure 2D–G) and the number of each application. Natural and internal systems differ in the total pollutant flow to their surfaces, with cities channelling a median of 30 GtCO_2_/y, three times higher than HVAC systems (10 GtCO_2_/y) and nearly an order of magnitude above HVAC filters (4 GtCO_2_/y) (Figure 3A). While this indicates that city environments offer the largest surface area to reach climate-relevant removal bounds, realizing this potential would require coating surfaces with removal technologies on a large scale and maintaining their performance over decades. HVAC filters, although receiving lower pollutant flow rates to their surfaces, provide more controlled conditions that may simplify fitting, monitoring, and maintenance; moreover, their regular replacement makes accessing the installed base more feasible, making them a potentially promising near-term option. Conversely, external airflows and small-surface-area internal systems (columns 5–8) show lower atmospheric pollutant flow rates to their surface, e.g., an average of 0.1 GtCO_2_/y for aeroplanes, 0.7 GtCO_2_/y for trains, 7 × 10^–3^ GtCO_2_/y for combustion systems, and 3 × 10^–7^ GtCO_2_/y for DAC systems, despite external airflows exhibiting higher atmospheric pollutant fluxes to their surfaces in Figure 2A–C.

**Figure 3:**
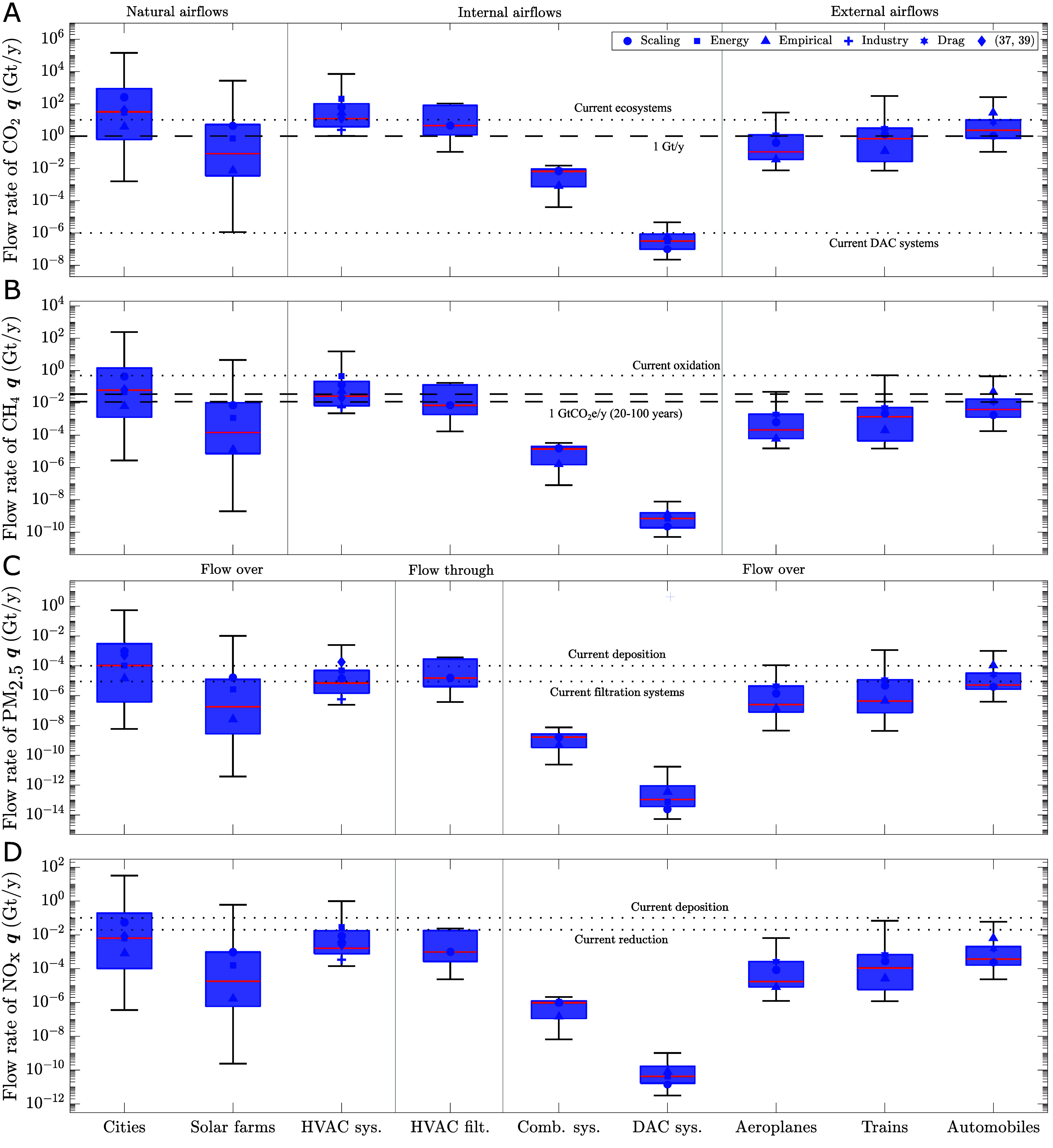
Atmospheric pollutant flow rates to the surfaces of global environments. Flow rates (***q***) of atmospheric (A) CO_2_, (B) CH_4_, (C) PM_2.5_, and (D) NO_*x*_ to the total existing surfaces in natural (e.g., cities, solar farms), internal (e.g., HVAC systems, HVAC filters, combustion systems, DAC systems), and external (e.g., aeroplanes, trains, automobiles) environments, with symbols representing the methods used to estimate flow rates. Horizontal lines mark estimated removal rates for atmospheric CO_2_, CH_4_, PM_2.5_, and NO_*x*_ due to ecosystems, oxidation, deposition, and reduction.^22,23,38,41,48,52,54^ Flow rates are derived using scaling (eqs 1–2), energy (eqs 5–6), empirical (eqs 3–4), industry (eqs 8–10), and drag (eq 7) approaches and Tables S1–S4.

For context, the global ecosystem acts as a carbon sink of approximately 10 GtCO_2_/y, primarily through photosynthesis and biomass accumulation, while upland soils remove roughly 0.03 GtCH_4_/y through methanotrophic oxidation.^48,52^ As shown in Figure 3A, these ecosystem removal rates provide a benchmark for the transport-limited pollutant flow rates to surfaces estimated here. For reference, the global vegetation surface area is approximately *O*(10^14^) m^2^,^53^ 2 orders of magnitude larger than the median city surface area considered here (*O*(10^12^) m^2^ estimated in Section S2). Therefore, although the median atmospheric delivery rates of CO_2_ and CH_4_ to city surfaces slightly exceed these global ecosystem sink rates, the total realizable removal would likely be lower due to the smaller city surface area and the dependence of removal on surface reactivity, residence time, and energy constraints.

#### Produced Surfaces

To approximate potential deployment rates, we scale atmospheric pollutant transport to yearly produced surfaces rather than to total existing surfaces. Because infrastructure is replaced or expanded gradually, flow rates to new surfaces decrease: CO_2_ transport to urban surfaces drops from 30 Gt/y for existing infrastructure to 0.2 Gt/y for new surfaces, while solar farms decline from 0.08 Gt/y to 0.03 Gt/y (Figure 3; Figure S2). This contrast arises from differing growth rates: solar capacity is expanding at about 20% per year, compared to approximately 4% annual growth in urban area (Table S2). These results indicate that although cities contain the largest absolute removal capacity, solar farms accumulate removal potential more rapidly, underscoring that deployment time scales, not just total capacity, are critical for planning implementation.

### Potential Removal Rates of Atmospheric Pollutants

#### CO_2_ Using Sorption

Having established the flow rate of CO_2_ to the surfaces of various natural and mechanical environments, we assess the transport-limited upper bound to identify which environments could in principle remove atmospheric CO_2_ from the atmosphere at rates exceeding the 1 GtCO_2_/y benchmark.^55^ The feasibility of applying surface-based technologies in these environments, including potential impacts on their primary function, is discussed in Section S4. The atmospheric CO_2_ flow rate exceeds 1 GtCO_2_/y for cities (IQR: 6 × 10^–1^ to 9 × 10^2^ GtCO_2_/y), solar farms (4 × 10^–3^ to 5 × 10^0^ GtCO_2_/y), HVAC systems (4 × 10^0^ to 1 × 10^2^ GtCO_2_/y), HVAC filters (1 × 10^0^ to 8 × 10^1^ GtCO_2_/y), aeroplanes (4 × 10^–2^ to 1 × 10^0^ GtCO_2_/y), trains (3 × 10^–2^ to 3 × 10^0^ GtCO_2_/y), and automobiles (7 × 10^–1^ to 1 × 10^1^ GtCO_2_/y), confirming that for these environments atmospheric transport is not the binding constraint relative to the 1 GtCO_2_/y benchmark (Figure 3A). However, achieving this theoretical upper bound would require coating *O*(10^12^) m^2^ surface areas, implying a large global-scale material production and long-term maintenance effort. If one only considers flow rates to annually produced surfaces (Figure S2A), which can provide an estimate for potential deployment rate or market penetration, feasible applications narrow to cities, solar farms, HVAC systems, and filters. For example, if sorbents were applied only to new building materials, the transition from annually produced surfaces to total existing surfaces would occur gradually over decades (Table S2).

The realization of the above transport-limited potentials is constrained by the fact that removal efficiencies are often suboptimal, and the deployment of sorbents across surface areas is limited by the necessity of a complete removal chain. For example, DAC systems using sorbents exhibit removal efficiencies of around 5 × 10^–2^ (based on Soeherman et al.,^27^ which reviews various CO_2_-removal technologies), giving a median removal potential of 2 × 10^0^ GtCO_2_/y for cities, 6 × 10^–1^ GtCO_2_/y for HVAC systems, and 1 × 10^–1^ GtCO_2_/y for automobiles with sorbent surfaces. If applications are reaction- or regeneration-limited, removal efficiencies may be lower. For example, if removal efficiency decreases by 2 orders of magnitude to 5 × 10^–4^ in natural and external airflows, the corresponding removal potentials decrease to approximately 2 × 10^–2^ GtCO_2_/y for cities and 1 × 10^–3^ GtCO_2_/y for automobiles. We can compare these potential removal rates with current DAC systems, which are removing around 10^3^ tCO_2_/y (based on McQueen et al.,^29^ which reviews DAC approaches in the US) or 10^–6^ GtCO_2_/y (Figure 3A), but have a smaller surface area.

For amine-functionalized solid sorbents used in DAC systems, typical working capacities are 1 to 3 mmol/g with regeneration cycles of approximately 15 to 85 min.^29^ Total system energy requirements, including sorbent regeneration and air handling, are typically 6 to 10 GJ per tCO_2_ removed. Scaling these operational requirements to the median sorbent removal potential estimated here (2 GtCO_2_/y for city surfaces) implies energy inputs on the order of 12 to 20 EJ/y, corresponding to several percent of current global energy consumption. Given these constraints, realized net removal would be substantially lower than the flow rate estimates, depending on sorbent cycling efficiency, energy availability, and proximity to sequestration infrastructure.

A potential removal rate of 2 GtCO_2_/y, proportional to the predicted median sorbent removal potential for city surfaces, corresponds to a potential surface removal rate constant *k*_surf_ ≈ 6 × 10^–4^ y^–1^ relative to the atmospheric burden of approximately 3200 GtCO_2_.^48^ This is small relative to the natural removal rate constant (0.01 to 0.02 y^–1^), indicating that removal at this scale would have a limited effect on atmospheric CO_2_ concentrations at the global scale. In contrast, the transport-limited upper bound removal potential of approximately 30 GtCO_2_/y, corresponds to *k*_surf_ ≈ 9 × 10^–3^ y^–1^, approaching the lower bound of the natural removal rate constant. At this scale, potential removal could perturb atmospheric concentrations, reducing concentration gradients driving surface fluxes and changing the transport-limited estimates over time. Achieving removal at this scale would require the entire urban surface area to be coated with sorbent materials operating at laboratory-scale efficiency, with each surface regenerated and the captured CO_2_ concentrated, compressed and sequestered, a chain of requirements that currently lies beyond demonstrated technological and economic feasibility.

#### CH_4_ Using Catalysis

Methane removal is more challenging due to its lower atmospheric concentration, but catalytic approaches can reach removal efficiencies of around 4 × 10^–2^ (based on Tsopelakou et al.,^30^ which reviews CH_4_ photocatalyst data). When this laboratory-scale removal efficiency is applied to the upper range of modeled CH_4_ flow rates (upper 5% of estimated values), the resulting transport-limited upper-bound removal potentials reach 10 and 1 GtCH_4_/y for cities and HVAC systems, respectively (Figure 3B), exceeding the estimated atmospheric oxidation rate of approximately 0.5 GtCH_4_/y,^52^ which is comparable to global CH_4_ emission rates of around 0.55 GtCH_4_/y.^49^ At ambient temperatures and concentrations, CH_4_ oxidation can be kinetics-limited and removal efficiencies may be lower than laboratory values in some applications. If removal efficiencies in cities decrease to 4 × 10^–4^, e.g., the upper-bound kinetics-limited removal potential in cities would decrease to approximately 1 × 10^–1^ GtCH_4_/y. Real-world constraints have led to varied pollutant reductions in field demonstrations of photocatalytic surfaces^56,57^ . For CH_4_, the upper-bound kinetics-limited potential removal rate for city surfaces of approximately 1 × 10^–1^ GtCH_4_/y corresponds to *k*_surf_ ≈ 0.02 y^–1^ relative to the atmospheric burden of approximately 5.4 GtCH_4_.^49^ This is smaller than the natural atmospheric oxidation rate constant of approximately 0.1 y^–1^, indicating that, under kinetics-limited conditions, surface-based removal could have a limited effect on atmospheric concentrations. Furthermore, this assumes sustained catalytic activity across all city surfaces at kinetics-limited catalytic efficiencies and ambient concentrations,^31^ and catalyst stability over deployment time scales under ambient conditions in applications is far from demonstrated.

Reported reaction probabilities for CH_4_ oxidation on catalytic surfaces are typically γ = *O*(10^–9^) per molecule–surface collision under ambient conditions,^31^ such that removal efficiency scales as γ*N*_coll_, where *N*_coll_ is the number of molecule–surface collisions per transit. A laboratory removal efficiency of 4 × 10^–2^ is therefore consistent with *N*_coll_ = *O*(10^7^) collisions in reactor geometries.^30^ In applications, *N*_coll_ is expected to be several orders of magnitude lower due to shorter residence times, although flow-through configurations (e.g., HVAC filters) could increase molecule–surface interactions relative to flow-over surfaces. Photocatalytic CH_4_ oxidation is further limited by low quantum efficiency, implying high photon flux requirements and increased energy demand.^33^ In HVAC systems, UV illumination would be required for activation, introducing an energy cost, although UV irradiation is already used in some systems for microbial control;^58^ outdoor systems could instead utilize sunlight, but remain constrained by low quantum efficiency and ambient CH_4_ concentrations.^25^ In the contexts considered here, removal could leverage existing airflow and energy sources (e.g., sunlight outdoors or UV in HVAC systems), so additional energy inputs are minimal and climate break-even is achievable. If artificial airflow or irradiation were required, net climate benefit could be negative. Moreover, all applications exceed $100 per tCO_2_e (see Table 1). Consistent with these constraints, prior studies report that photocatalytic CH_4_ removal can be cost-ineffective and may be climate-detrimental at ambient concentrations.^33^ Removal efficiency, energy balance, and cost per tonne are strongly application-specific; the present analysis instead provides an upper bound on atmospheric transport to surfaces and does not imply technical, cost, or ultimate viability.

**Table 1:** Material and Application Cost of Surface-Based Atmospheric Pollutant Removal in Global Environments^a^

environment	removal rate (t/y)	surface area (m^2^/y)	technology cost ($/y)	removal cost ($/t)	removal cost ($/tCO_2_e)
Carbon Dioxide (CO_2_)					
cities	[5 × 10^2^, 2 × 10^10^]	[5 × 10^9^, 1 × 10^12^]	[8 × 10^10^, 5 × 10^13^]	[3 × 10^3^, 2 × 10^8^]	[3 × 10^3^, 2 × 10^8^]
solar farms	[2 × 10^2^, 1 × 10^10^]	[5 × 10^7^, 3 × 10^11^]	[8 × 10^8^, 1 × 10^13^]	[1 × 10^3^, 4 × 10^6^]	[1 × 10^3^, 4 × 10^6^]
HVAC systems	[5 × 10^5^, 5 × 10^9^]	[3 × 10^9^, 6 × 10^10^]	[5 × 10^10^, 3 × 10^12^]	[6 × 10^2^, 1 × 10^5^]	[6 × 10^2^, 1 × 10^5^]
HVAC filters	[5 × 10^5^, 1 × 10^9^]	[2 × 10^7^, 8 × 10^10^]	[3 × 10^8^, 4 × 10^12^]	[6 × 10^2^, 4 × 10^3^]	[6 × 10^2^, 4 × 10^3^]
Methane (CH_4_)					
cities	[2 × 10^0^, 4 × 10^7^]	[5 × 10^9^, 1 × 10^12^]	[8 × 10^10^, 4 × 10^13^]	[1 × 10^6^, 4 × 10^10^]	[1 × 10^4^, 5 × 10^8^]
solar farms	[8 × 10^–1^, 4 × 10^7^]	[5 × 10^7^, 3 × 10^11^]	[8 × 10^8^, 1 × 10^13^]	[3 × 10^5^, 1 × 10^9^]	[4 × 10^3^, 1 × 10^7^]
HVAC systems	[4 × 10^3^, 2 × 10^7^]	[3 × 10^9^, 6 × 10^10^]	[4 × 10^10^, 3 × 10^12^]	[2 × 10^5^, 1 × 10^7^]	[2 × 10^3^, 1 × 10^5^]
HVAC filters	[2 × 10^3^, 2 × 10^6^]	[2 × 10^7^, 8 × 10^10^]	[3 × 10^8^, 4 × 10^12^]	[2 × 10^5^, 2 × 10^6^]	[2 × 10^3^, 2 × 10^4^]

a Values in brackets represent minimum–maximum ranges. The upper-bound potential removal rate of atmospheric CO_2_ and CH_4_ per year using representative laboratory-scale removal efficiencies of 5 × 10^–2^ for CO_2_ sorbents and 4 × 10^–2^ for CH_4_ catalysis, surface area produced per year, technology material and application cost per year based on material and application cost per square meter,^16,25,28,32^ material and application cost per tonne of atmospheric CO_2_ and CH_4_ removed, and material and application cost per tonne of CO_2_e removed for different natural and internal flows. Data for these natural and internal flows are divided into cities, solar farms, HVAC systems, and filters that have potential removal rates of atmospheric CO_2_ and CH_4_ that exceed the 1 GtCO_2_e/y target (20-year GWP) in Figure 3.

#### PM_2.5_ Using Filtration and Oxidized Forms of NO_*x*_ via Deposition

PM_2.5_ is captured by HVAC filters which achieve removal efficiencies of around 9.9 × 10^–1^ (based on Schroth,^35^ which compares various filter classes), such that the average removal potential is 10^–5^ GtPM_2.5_/y (Figure 3C). Atmospheric removal rates approach 9 × 10^–6^ GtPM_2.5_/y, with estimates of 100 million filters processing 100 m^3^/h at 99% removal efficiency (based on Lowther et al.,^36^ which compares PM removal across rooms in China). These removal efficiencies represent operational averages that depend on filter replacement cycles and are higher than those of sorption or catalyst technologies. By contrast, PM_2.5_, as well as the oxidized forms of NO_*x*_, are captured when they interact with surfaces through deposition. As shown in Figure 3C,D, we compare dry deposition rates of 1 × 10^–4^ GtPM_2.5_/y and 1 × 10^–1^ GtNO_*x*_/y with atmospheric pollutant flow rate estimates to urban surfaces (based on Wu et al.,^38^ which measured deposition of particulate matter during summer in Beijing, and Dore et al.,^54^ which modeled deposition of sulfur and nitrogen across the UK), contributing around 1 to 10% of global deposition.

Air pollution is one of the leading environmental risk factors for premature mortality, with ambient air pollution contributing to approximately 4.2 million premature deaths annually and household air pollution contributing approximately 2.9 million, combining to approximately 7 million deaths per year worldwide.^9^ Global anthropogenic primary carbonaceous aerosol emissions in 2017 were approximately 22 Tg/y, representing a lower bound on total PM_2.5_ emissions, and NO_*x*_ emissions were approximately 122 Tg/y.^59^ The estimated median potential removal rates of 1 × 10^–4^ GtPM_2.5_/y and 2 × 10^–4^ GtNO_*x*_/y correspond to approximately 0.45% and 0.16% of these global anthropogenic emission totals, respectively. Assuming steady-state conditions and that concentration scales proportionally with emissions, these removal rates correspond to concentration reductions of approximately 0.11 μg/m^3^ for PM_2.5_ and 0.04 μg/m^3^ for NO_2_ at a typical urban mean concentration of 25 μg/m^3^ for each pollutant. Using exposure–mortality relationships of 2.8% per 10 μg/m^3^ for PM_2.5_ ^60^ and 4% per 10 μg/m^3^ for NO_2_,^61^ these concentration reductions correspond to changes in pollutant-associated mortality of approximately 0.03% and 0.02%, respectively. These estimates are simplified and do not account for atmospheric chemistry, spatial heterogeneity, or secondary aerosol formation; nonetheless, they imply that potential removal at the scales estimated here would correspond to small changes in pollutant-associated mortality at the global scale.

### Material and Application Costs

Finally, we estimate the potential material and application cost of scaling CO_2_-sorption and CH_4_-catalyst technologies, highlighting their capacity for atmospheric removal if deployed in different environments, with reported material and application costs ranging from $16 to $50 per m^2^ for CO_2_ sorbents (based on values given in Figure 28 of Sanz-Pérez et al.^16^ and Figure 7 of Kulkarni et al.^62^) and $15 to $43 per m^2^ for CH_4_ catalysts (based on values given in the SI of Randall et al.^25^ and Hickey et al.^32^). These values exclude maintenance, regeneration energy, replacement frequency, performance degradation, and operational energy inputs (e.g., UV illumination for photocatalysts, which may dominate system-level costs in internal environments; increased fan power due to pressure-drop penalties is expected to be small). Total costs will vary with the application, and a techno-economic analysis would be required for each deployment scenario. The material and application costs of $16 to $50 per m^2^ for CO_2_ sorbents exclude the infrastructure required for the capture-to-storage chain. Capture and regeneration costs reported for DAC systems typically range from $88 to $600 per tCO_2_ removed, which is at or below our lower bound estimate in Table 1, while compression and sequestration can add an additional $15 to $21 per tCO_2_, excluding transport costs which are site-specific.^63^ Applying these cost ranges to the capture potentials estimated here implies additional system costs of around $103 to $621 per tCO_2_ potentially removed, or annual expenditures on the order of $2 × 10^11^ to $1.2 × 10^12^ per year if potential removal were sustained at 2 GtCO_2_/y, which is comparable to up to around 1% of current global GDP.

The costs of $15 to $43 per m^2^ reported here for CH_4_ oxidation catalysts include materials and application, with materials representing a smaller fraction of the total, where periodic maintenance adds approximately 11% to the cost annually.^25^ . In contrast, operational energy inputs can dominate system costs depending on the activation mechanism. For outdoor environments such as cities or transportation infrastructure, photocatalytic activation may be driven by natural radiation and therefore require little energy input. For enclosed environments such as HVAC systems, UV illumination may be required at irradiance levels of approximately 10 to 100 W/m^2^.^30^ If applied across the global HVAC ventilation median surface area (approximately 5 × 10^11^ m^2^ estimated in Section S2), the required electrical power would be approximately 5 to 50 TW, corresponding to an annual electricity demand of approximately 4 × 10^4^ to 4 × 10^5^ TWh/y, which exceeds current global electricity production. At an electricity cost of $0.08 per kWh, this corresponds to an annual electricity expenditure of $3.5 to $35 trillion, or approximately $2100 to $21,000 per tCO_2_e potentially removed (20-year GWP) at the median flow rate of 2 × 10^–2^ GtCH_4_/y, greater than the lower bound system cost of approximately $2000 per tCO_2_e estimated from materials and application alone in Table 1. However, in some indoor environments, UV illumination is already deployed for air treatment (e.g., microbial control), and photocatalytic systems could leverage this existing infrastructure depending on wavelength compatibility and required irradiance, reducing installation and energy costs. This highlights that energy inputs can become a dominant cost component for indoor configurations, and that the feasibility of catalytic CH_4_ removal is sensitive to the application and whether artificial UV illumination is required.

To evaluate cost-efficiency, we compare these material and application cost estimates with a benchmark of $100 per tCO_2_e potentially removed (20-year GWP).^55^ We adopt the 20-year GWP to provide an upper-bound estimate of CO_2_-equivalent benefit per tonne CH_4_ removed, consistent with the upper-bound transport-limited framework of this study, noting that CH_4_ has an atmospheric lifetime of approximately 12 years and its warming impact is concentrated in the first few decades after emission. Sensitivity to alternative climate metrics is discussed in Section S7. We assume a removal efficiency of 5 × 10^–2^ for CO_2_ sorbents^27^ and 4 × 10^–2^ for CH_4_ catalysts.^30^ For each application in Table 1, we calculate the material and application cost per tonne of CO_2_e removed by dividing the estimated material cost of sorbent or catalyst technologies by the corresponding CO_2_ or CH_4_ removal rate. These estimates assume a single installation cycle and exclude costs associated with replacement frequency (discussed in Section S4), regeneration energy, and energy penalties. The reported values should therefore be interpreted as order-of-magnitude materials and application screening estimates rather than full lifecycle costs. All estimates across natural and internal environments exceed the $100 per tCO_2_e removed target. However, HVAC filters achieve potential material and application costs as low as $600 per tCO_2_ removed (range: $6 × 10^2^ to $4 × 10^3^ per tCO_2_ removed) for sorption, and as low as $2000 per tCO_2_e removed ($2 × 10^3^ to $2 × 10^4^ per tCO_2_e removed) for CH_4_-catalyst technologies (Table 1). This lower material and application cost arises from the high pollutant flux to HVAC filter surfaces (e.g., an average of 0.003 mol/(m^2^s) for CO_2_, Figure 2C), which reduces the surface area requirement per unit of pollutant removal. The feasibility of integrating removal technologies with periodic replacement and its effect on material and application cost is discussed in Section S4.

## Discussion

The potential for removing atmospheric pollutants, as a mitigation strategy for climate change and public health crises, has yet to be fully explored. Surface-based removal technologies have been proposed as a potential approach, with examples including sorbents for CO_2_ capture,^29^ catalysts to reduce VOCs and NO_*x*_,^64^ and HEPA filters for PM removal.^20^ CO_2_ sorbent materials have been demonstrated at laboratory or pilot scale, e.g., in DAC,^29^ sorbent filters within ventilation systems,^65^ or as carbonation coatings applied to buildings,^66^ and could in principle be integrated into wider infrastructure. Unlike carbonation coatings, in which captured CO_2_ is mineralized at the surface for a single application, sorbent filters could undergo periodic regeneration, but the captured CO_2_ must then be collected, compressed, transported, and sequestered to achieve climate benefits. Thus, while our analysis identifies large theoretical capture potentials based on atmospheric transport to surfaces, realizing sustained removal at scale requires integration of regeneration, energy, transport, and storage infrastructure and is therefore constrained by system-level energy and economic considerations.

Recent field deployments show photocatalytic systems achieving NO_*x*_ removal of up to 66% at street level and 82% on wall surfaces under ambient conditions. In tunnel environments, reductions of up to 23% have been reported under a light intensity of approximately 20 W/m^2^, air velocities of approximately 0.4 m/s, and relative humidity of 40 to 70%.^56^ These values represent near-surface removal efficiencies and are used here to define an upper-limit removal potential within the transport-based framework. However, Russell et al.^56^ conclude that outdoor deployments of TiO_2_ coatings often yield ambient concentration reductions of around 2% in the intermediate vicinity of treated surfaces. In addition, the formation of secondary species has been observed in some studies, such that air-quality impacts depend on surface reactivity and atmospheric chemistry. Ma et al.^57^ discuss the potential of photocatalytic systems to deliver atmospheric pollutant reductions outside the laboratory in cities, for a variety of species including NO_*x*_, VOCs, and O_3_. We apply our methodology to O_3_ in Section S5 and discuss a relevant real-world example of catalytic pollutant removal.

In forced internal environments such as HVAC systems, additional removal technologies could increase pressure drop and therefore energy consumption. Integrating catalysts into HVAC duct walls or filters is anticipated to have minimal impact on pressure drop (and could be accommodated through filter design or grade adjustments), whereas introducing additional flow-through removal structures (e.g., supplementary filters or catalytic monoliths) could lead to increases. In Europe, HVAC systems consume approximately 313 TWh/y of electricity;^67^ scaled to global consumption, with Europe representing approximately 10% of global electricity demand, this implies global HVAC electricity consumption of approximately 3100 TWh/y, equivalent to approximately 0.6 GtCO_2_/y at a grid carbon intensity of 0.2 kgCO_2_/kWh.^68^ Only a fraction of this energy is associated with air movement (fan power), which is typically around 20% of total HVAC electricity consumption. For example, if pollutant removal systems increased HVAC pressure drop by approximately 10%, fan electricity consumption would increase proportionally, corresponding to an additional 62 TWh/y or 0.012 GtCO_2_/y globally. For comparison, the CH_4_ removal potentials estimated here for HVAC filters correspond to approximately *O*(10^–4^) to *O*(10^0^) GtCO_2_e/y (20-year GWP). This comparison suggests that pressure-drop penalties could offset the climate benefit of additional removal technologies at low removal efficiencies; however, higher removal rates could remain climate-positive. Integrating surface-based pollutant removal technologies into HVAC systems could build upon their existing capabilities for PM and VOC removal,^20,69^ enhancing overall cost-effectiveness and scalability. A similar integration of technologies was suggested during the COVID-19 pandemic, when HVAC systems were adapted to mitigate airborne viruses, using technologies such as UV-C light and antimicrobial coatings.^58,70^ Furthermore, HVAC filters are regularly replaced, potentially making the maintenance of any surface-based removal technologies straightforward and allowing their performance to be sustained over time. A cost and climate break-even analysis of these additional removal technologies in HVAC systems is left for future work.

For context, using a laboratory removal efficiency we estimate a median potential removal rate of CO_2_ with sorbents using all city surfaces (2 GtCO_2_/y), corresponding to around 5% of global anthropogenic CO_2_ emissions (40 Gt/y).^48^ For CH_4_, the median potential removal rate with catalysis on all city surfaces (2 × 10^–3^ GtCH_4_/y) is around 0.4% of the estimated total natural atmospheric CH_4_ sink of approximately 0.5 GtCH_4_/y,^52^ and about 0.3 to 0.4% of global CH_4_ emission rates of around 0.55 GtCH_4_/y.^49^ For NO_*x*_, the median potential removal rate with catalysis on all city surfaces (2 × 10^–4^ GtNO_*x*_/y) corresponds to around 0.2% of NO_*x*_ emission rates of around 0.12 GtNO_*x*_/y.^59^ However, the removal potentials estimated here with laboratory removal efficiencies represent transport-limited upper bounds rather than demonstrated technological performance. Achieving real-world removal would require catalytic systems capable of sustained operation under atmospheric conditions, together with deployment across large surface areas. Current catalytic technologies typically exhibit lower reaction rates under atmospheric conditions and may require significant energy input.^30,33,56^ Such removal rates are therefore not currently achievable at scale and would require substantial technological advances before they could be realized in practice.

If potential removal rates approach the magnitude of natural sinks or anthropogenic emissions, then atmospheric concentrations would decrease, reducing concentration-driven flow rates to surfaces. While a well-mixed box model provides an estimate of concentration evolution, spatial heterogeneity, boundary-layer dynamics, and global circulation need to be integrated into atmospheric transport models. Future work will couple surface-based removal into pollutant transport and climate models to evaluate regional transport limitations, feedbacks with natural sinks, and atmospheric responses. A full assessment must also account for regeneration energy, replacement frequency, energy consumption, and performance degradation. These factors are important for dilute pollutants where large air volumes must be processed and will determine whether a given technology achieves climate break-even. Future work must integrate these engineering inputs with more specific system-level modeling to determine the cost and climate benefit for the promising environments identified here for atmospheric pollutant removal. Application-specific removal efficiencies measured under characteristic flow velocities, pollutant concentrations, humidity, and contact times are not currently available across the range of pollutants and applications considered here; characterizing these values represents an important direction for future work.

Scaling surface-based atmospheric pollutant removal technologies presents several additional challenges that must be addressed in future research, including material degradation, surface contamination, and infrastructure integration. Sorption systems generally maintain predictable CO_2_ removal efficiencies that decline as sorbent materials become saturated,^29^ whereas the catalytic removal of CH_4_ is more variable,^30,31^ and can fluctuate under varying conditions (e.g., relative humidity, light intensity). Advances in materials and reaction mechanisms can enhance these removal efficiencies and broaden the applicability of catalysis. However, large-scale deployment of catalysts may result in undesirable reactions, such as the partial oxidation of CH_4_ yielding formaldehyde,^71^ or the reduction of NO_*x*_ producing N_2_O.^72^ The distribution of such products can vary depending on catalyst composition, morphology, humidity, and pollutant mixtures. Mitigation measures include tailoring catalysts to favor complete oxidation of CH_4_ to CO_2_ and H_2_O while suppressing partial oxidation pathways,^71^ immobilizing catalysts on durable supports to minimize particle release,^73^ and operating under controlled temperature–humidity conditions to reduce N_2_O formation from NO_*x*_ reduction.^72^ In HVAC systems, catalytic removal could be combined with downstream filtration to capture any harmful intermediates before air is recirculated. Additional risks, such as catalyst poisoning by sulfur or siloxane species,^74^ can be mitigated via upstream prefiltration and periodic regeneration of catalysts.

These findings highlight the potential for integrating surface-based atmospheric pollutant removal technologies into urban and industrial systems to support climate action, while noting that technical advances are required to realize this potential.

## Supplementary Material


