# Supplementary material for: Evaluating the Scale of Natural and Mechanical Airflows for Surface-Based Atmospheric Pollutant Removal
Source: Environ Sci Technol. 2026 Jul 24;60(31):21609–21. doi: 10.1021/acs.est.5c16697 (PMC13472194; doi:10.1021/acs.est.5c16697)
Supplement: Supplementary file 1 [file es5c16697_si_001.pdf]

Supporting information for: Evaluating the scale of natural and mechanical airflows for surface-based atmospheric pollutant removal

Samuel D. Tomlinson,[*,†,‡,¶] Aliki M. Tsopelakou,[†,‡] Tzia M. Onn,[†,‡] Steven R. H. Barrett,[†] Adam M. Boies,[*,†,‡,§] and Shaun D. Fitzgerald[*,†,‡]

†*Department of Engineering, University of Cambridge, Cambridge, CB2 1PZ, UK*

‡*Centre for Climate Repair, Cambridge, CB3 0WA, UK*

¶*School of Computing and Mathematical Sciences, University of Greenwich, London, SE10 9LS, UK*

§*Department of Mechanical Engineering, Stanford University, Stanford, California, 94305, USA*

E-mail: sdt50@cam.ac.uk; aboies@stanford.edu; sdf10@cam.ac.uk

S1 Atmospheric concentrations and diffusivities of pollutants

Understanding the fluxes and flow rates of greenhouse gases (GHGs) and atmospheric pollutants across various environments requires knowledge of their atmospheric concentrations (c), as well as their diffusivities (D). Table S1 presents ranges of typical atmospheric concentrations and diffusivities of carbon dioxide (CO_2), methane (CH_4), nitrous oxide (N_2O), particulate matter ($PM_{2.5}$), sulphur dioxide (SO_2) and nitrogen oxides (NO_x) in the atmosphere. We have included ranges as atmospheric concentrations can vary inside natural and mechanical environments due to emissions, removal and dispersion.[1] We use these six examples to display our results, however, the methods used are general and one would only need

to change c and D to model a different atmospheric pollutant.

S2 Estimating parameters for natural and mechanical airflows

This section describes the approach used to collect and estimate the parameter ranges in Tables S2–S4. We provide minimum, mean and maximum values for the length, surface area, velocity, total number and produced number for each example of natural (Section S2.1), internal (Section S2.2) and external (Section S2.3) flow. Additionally, we include estimates for heat flux and temperature difference in Section S2.1; power, specific fan power (SFP), cubic feet per minute (CFM) per person, occupancy, CFM per square meter, building floor area, air changes per hour (ACH) and building volume in Section S2.2; and drag force and drag coefficient in Section S2.3.

Parameter values were primarily derived from literature and measurement datasets. Where sufficient data were available (e.g., city velocity distributions, Section S2.1.1), we directly estimated minimum, mean and maximum values. For parameters with limited data, representative literature values were selected for the mean, and a conservative range, typically spanning an order of magnitude, was applied to capture uncertainty. These triangular distributions were substituted into the atmospheric pollutant flux and flow rate equations (Section S3). The resulting distributions of outcomes are summarised in Figs. 1–3 of the main text, with mean values and box-and-whisker plots used to illustrate variability across methods.

S2.1 Natural flows

S2.1.1 Cities

The minimum and maximum lengths of cities are assumed to vary between 7×10^3 m and 5×10^4 m. In Bettencourt *et al.*,[2] the minimum length is reported as 7×10^3 m, with a corresponding minimum area of $(7 \times 10^3)^2 = 5 \times 10^7$ m^2 for Las Palmas, and a maximum length of 5×10^4 m with an area of 3×10^9 m^2 for London. Wind speeds in cities, influenced by various local factors,[1] typically range from 1 to 15 m/s. Measurements by Holzworth *et al.*[3] in several U.S. locations indicate a minimum wind speed of 1 m/s and a maximum of 13 m/s. The global number of cities is estimated to be between 1×10^3 and 1×10^4, depending on definitions based on size or population. In Angel *et al.*,[4] the number of cities worldwide is estimated at 3646. They further predict that urban land cover will increase from 3×10^5 km^2

Table S1: Assumed ambient concentration ranges (mol/m^3) and molecular diffusivities (m^2/s) of CO_2, CH_4, N_2O, $PM_{2.5}$, SO_2 and NO_x, used for modelling atmospheric pollutant behaviour.[8–13]

Species	Ambient concentration (mol/m^3)	Diffusivity (m^2/s)
CO_2	$[2 \times 10^{-2}, 4 \times 10^{-2}]$	2×10^{-5}
CH_4	$[6 \times 10^{-5}, 1 \times 10^{-4}]$	2×10^{-5}
N_2O	$[8 \times 10^{-6}, 2 \times 10^{-5}]$	2×10^{-5}
$PM_{2.5}$	$[1 \times 10^{-7}, 1 \times 10^{-6}]$	1×10^{-8}
SO_2	$[2 \times 10^{-7}, 5 \times 10^{-6}]$	1×10^{-5}
NO_x	$[2 \times 10^{-6}, 8 \times 10^{-6}]$	2×10^{-5}

to 1.2×10^6 km^2 over 50 years, suggesting that around 300 new cities are produced annually using Bettencourt *et al.*[2] We assume the heat flux in cities ranges from 10 W/m^2 to 600 W/m^2, supported by evidence in Allen *et al.*,[5] where average values range from 10 W/m^2 in smaller cities to 600 W/m^2 in New York. Temperature differences between city centres and surrounding areas can range from 1°C to 10°C. In Erell *et al.*,[6] temperature differences of this magnitude were observed between urban street canyons and suburban areas in Adelaide. The deposition velocity varies from 0.001 m/s to 0.01 m/s and the friction velocity varies from 0.1 m/s to 1 m/s in Giardina *et al.*[7]

Building lengths are assumed to range from 6 to 100 m, with surface areas varying from 40 to 1×10^4 m^2. In London, Steadman *et al.*[14] reports building surface areas between 40 m^2 for smaller buildings and 10000 m^2 for larger ones. Assuming walls are approximately square (surface area $\sim$ length^2), this corresponds to building lengths from 6 m for small residential units to 100 m for large commercial structures. The current number of buildings worldwide is estimated to range from 3×10^7 to 1×10^8, with 2 to 8 million new buildings produced annually. These estimates align with city building density assumptions of 10 to 40 buildings per hectare,[15] resulting in a lower bound of 3×10^7 and an upper bound of 1×10^8. Based on urban expansion rates from Angel *et al.*,[4] this yields 2 to 8 million new buildings annually.

S2.1.2 Solar farms

We assume that small solar farms typically span around 300 m in length and cover an area of 1×10^5 m^2, while large solar farms can extend up to 7000 m in length, covering an area of 5×10^7 m^2. These ranges are supported by Woolway *et al.* and Jones *et al.*,[16,17] which describe small installations covering 1×10^5 m^2 and the largest solar farms reaching up to 5×10^7 m^2. Globally, the number of solar farms is estimated to be between 1×10^3 and 2×10^4, with 500 to 5000 new farms being established annually. According to Hossain *et al.*,[18] the global installed capacity of solar farms reached 1400 GW in 2023, up from 1100 GW in 2022. Assuming an average capacity of 100 MW per farm, this translates to approximately 20000

Table S2: The minimum, mean and maximum parameter values for the length (m), surface area (m^2), velocity (m/s), total number (–), produced number (–), heat flux (W/m^2), surface area (m^2), temperature difference (K) and power (W) are shown. We consider natural flows over cities,[2–6] buildings,[3,4,14,15] solar farms[3,16–20] and solar panels.[3,16,17,21]

Environment	Parameter	Ref.	Min.	Mean	Max.	Parameter	Ref.	Min.	Mean	Max.
Cities	Length (m)	2	7×10^3	1×10^4	5×10^4	Total number (–)	4	1×10^3	5×10^3	1×10^4
	Surface area (m^2)	2	5×10^7	1×10^8	3×10^9	Produced number (–)	4	100	200	400
	Velocity (m/s)	3	1	4	15	Heat flux (W/m^2)	5	1×10^1	1×10^2	6×10^2
	Temperature difference (°C)	6	1	3	10					
Buildings	Length (m)	14	6	30	100	Total number (–)	15	1×10^7	5×10^7	1×10^8
	Surface area (m^2)	14	40	1×10^3	1×10^4	Produced number (–/y)	4	2×10^6	4×10^6	8×10^6
	Velocity (m/s)	3	1	4	15					
Solar farms	Length (m)	16	300	750	7×10^3	Total number (–)	18	2×10^3	1×10^4	5×10^4
	Surface area (m^2)	16	1×10^5	5×10^5	5×10^7	Produced number (–)	18	500	2×10^3	5×10^3
	Velocity (m/s)	3	1	4	15	Heat flux (W/m^2)	19,20	1	5	15
	Temperature difference (°C)	19,20	1	2	5					
Solar panels	Length (m)	21	1	4	10	Total number (–)	18	1×10^7	5×10^7	7×10^8
	Surface area (m^2)	16	1	16	100	Produced number (–/y)	18	1×10^7	5×10^7	1×10^8
	Velocity (m/s)	3	1	4	15					

solar farms worldwide and about 3000 new farms annually. We assume wind speeds over solar farms are consistent with those reported for cities in Holzworth *et al.*[3] We estimate the heat flux across solar farms to range from 1 to 30 W/m^2, with temperature differences varying between 1°C and 8°C. In Glick *et al.*,[19] a heat flux of approximately 1 W/m^2 is reported, while Yang *et al.*[20] suggests a net radiation of around 100 W/m^2, implying a heat flux of up to 30 W/m^2. Both studies report temperature differences of 1°C to 8°C.

Solar panel dimensions are assumed to range from 1 m for small panels to 10 m for large installations. In Saad *et al.*,[21] panel dimensions are generally around 2 m; however, we also account for the potential stacking of panels in large farms. Consequently, panel surface areas are expected to vary from 1 m^2 to 100 m^2. The total global number of solar panels is estimated to be between 5×10^7 and 5×10^8, with new installations ranging from 1×10^7 to 4×10^8. Based on the panel size and farm capacities in Woolway *et al.* and Jones *et al.*,[16,17] we estimate that the average solar farm has about 5×10^4 panels, giving a total of 5×10^8 panels globally, with about 1×10^8 new panels produced each year.

S2.2 Internal flows

S2.2.1 HVAC systems

Average duct diameters are assumed to range from approximately 0.2 to 2 m. In large commercial buildings, Fisk *et al.*[22] reports that smaller ducts have cross-sectional dimensions

Table S3: The minimum, mean, and maximum parameter values for the diameter (m), surface area (m^2), velocity (m/s), total number (–), produced number (–), specific fan power ($W/(m^3/s)$), CFM per person (ft^3/min), number of people (–), CFM per unit area ($ft^3/min/m^2$), building floor area (m^2), air changes per hour (1/h), building volume (m^3), length (m), pleat number (–) and efficiency (%) for various systems. We consider HVAC systems,[22–29] combustion systems,[22,24,25,30–32] DAC systems[22,24,25,33,34] and HVAC filters.[35–37]

System	Parameter	Ref.	Min.	Mean	Max.	Parameter	Ref.	Min.	Mean	Max.
HVAC	Diameter (m)	22	0.2	0.7	2.0	CFM per person (ft^3/min)	26	20	35	50
	Surface area (m^2)	22	300	1×10^3	2×10^3	People (–)	27	2×10^9	4×10^9	5×10^9
	Velocity (m/s)	23	2	3.5	5	CFM per m^2 ($ft^3/min/m^2$)	28	0.1	1.5	3
	Total number (–)	15	2×10^8	5×10^8	1×10^9	Floor area (m^2)	28	1×10^{11}	4×10^{11}	1×10^{12}
	Produced number (–)	4	1×10^7	2×10^7	3×10^7	Air changes per hour (1/h)	29	0.5	3	5
	Power (W)	24	1×10^8	1×10^{10}	1×10^{11}	Volume (m^3)	28	6×10^{11}	1×10^{12}	2×10^{12}
	Specific fan power ($W/(m^3/s)$)	25	0.2	1.5	2.5					
Combustion	Diameter (m)	22	0.2	0.7	2.0	Produced number (–)	32	100	500	1×10^3
	Surface area (m^2)	30	50	275	500	Power (W)	24,32	2×10^5	7×10^5	1×10^6
	Velocity (m/s)	31	10	20	30	Specific fan power ($W/(m^3/s)$)	25	0.2	1.5	2.5
	Total number (–)	32	1×10^4	2×10^4	5×10^4					
DAC	Diameter (m)	22	0.2	0.7	2.0	Produced number (–)	34	10	50	150
	Surface area (m^2)	38	100	300	500	Power (W)	24,34	400	600	800
	Velocity (m/s)	33	1	3	6	Specific fan power ($W/(m^3/s)$)	25	0.2	1.5	2.5
	Total number (–)	34	20	100	300					
Filters	Length (m)	35	0.01	0.1	0.3	Efficiency (%)	35	97.0	99.0	100.0
	Surface area (m^2)	36	0.2	20	200	Total number (–)	37	5×10^8	1×10^9	2×10^9
	Velocity (m/s)	33	0.09	0.1	0.4	Produced number (–/y)	37	1×10^8	3×10^8	4×10^8

of 15–30 cm, while larger ducts can reach several meters. We estimate duct surface areas to vary from 300 to 2000 m^2. In Fisk *et al.*,[22] the authors examine HVAC systems in both small and large buildings, with duct surface areas ranging from 220–710 m^2 in smaller buildings and 1400–3400 m^2 in larger ones. Air velocity in HVAC ducts is typically estimated to fall between 2 and 5 m/s. In Fisk *et al.*,[23] maximum air velocities measured in their systems ranged from 0.8 to 3 m/s, consistent with our assumed range. Our analysis uses the global building counts and new construction rates discussed in Section S2.1.1. The power consumption of HVAC systems is assumed to range from 100 MW to 100 GW. According to Knight *et al.*,[24] 313 TWh are consumed annually in Europe alone, which translates to approximately $313 \times 10^{12}/8760 = 4 \times 10^{10}$ W, fitting within our estimated range. The deposition velocity varies from 10^{-6} m/s to 1 m/s in Sippola *et al.*[39]

We assume the specific fan power varies from 0.5 to 2.5 $W/(m^3/s)$, aligning with field measurements in Merzkirch *et al.*,[25] where specific fan power ranged from 0.23 to 1.9 $W/(m^3/s)$. Ventilation capacity per person is assumed to range from 20 to 50 cubic feet per minute (CFM). A reported value of 35 CFM per person for medium-sized U.S. offices in Eto *et al.*[26] is within this range. The global working population is estimated at 2 to 5 billion people,

with a midpoint of 4 billion, or approximately 50% of the global population, aligning with estimates from Herrmann *et al.*[27] For ventilation per floor area, we assume values from 0.5 to 4 CFM per m^2, consistent with measurements in Persily *et al.*,[28] where the CFM per m^2 varied from 0.6 to 3. We estimate the global floor area at 1×10^{11} to 1×10^{12} m^2, validated by typical floor areas of 100 to 3000 m^2 in Persily *et al.*[28] multiplied by the estimated building count ($5 \times 10^8 \times 3 \times 10^3 = 1.5 \times 10^{12}$ m^2). Air changes per hour (ACH) are assumed to range from 0.5 to 5. While Memarzadeh *et al.*[29] reports ACH values of 4 to 12, the higher end generally applies to hospital environments. For general building volumes, we estimate a range from 6×10^{11} to 2×10^{12} m^3. This estimate is validated by multiplying the global floor area from Persily *et al.*[28] by the building count and an assumed ceiling height of 2 m, yielding 2×10^{12} m^3.

Note that we could further divide these HVAC systems into buildings, clean rooms and data centres, which perturbs the representative duct dimensions, velocities and total/produced numbers that we would have to find in the literature or using industry standards like SFP, CFM or ACH. However, this does not introduce any new physics to these internal flows and so is neglected from our analysis.

S2.2.2 Combustion systems

Fossil fuel combustion chambers require robust ventilation systems to maintain the high airflow essential for combustion. We assume the duct diameters in these systems align with the range specified in Section S2.2.1. The surface area of these systems is estimated to range between 50 and 800 m^2, supported by Hale *et al.*,[30] which reports power plant sizes ranging from 200 to 800 m. Assuming a typical duct diameter of 1 m, this would imply similar duct lengths within our assumed range. Higher airflow rates than standard HVAC systems, estimated between 10 and 30 m/s, are necessary to ensure efficient fuel combustion. As noted in Zhang *et al.*,[31] an airflow velocity of 20 m/s is common, sitting centrally within this range. Globally, we estimate the number of power plants ranges from 1×10^4 to 5×10^4, with annual additions of 100 to 1000 new plants. The operational estimate of 1×10^4 plants given by Carbon Brief[32] supports the lower end of this spectrum. Based on operational power data from the past decade, which spans 1.6×10^6 to 1.8×10^6 MW, we estimate around 120 new power plants are added each year. Using these figures alongside the energy and specific fan power metrics from Section S2.2.1, we estimate that the power required for ventilation in these systems ranges from 2×10^5 to 1×10^6 W. Specific fan power is assumed to vary between 0.5 and 2.5 W/(m^3/s), consistent with similar industrial applications.

S2.2.3 Direct air capture (DAC) systems

We maintain the mean diameter of DAC ventilation systems within the range of 0.2–2 m, as specified in Section S2.2.1. The estimated surface area of DAC systems ranges from 100 to 500 m^2, consistent with dimensions observed in large DAC plants, which measure around 100 m in length.[38] Velocity estimates, based on airflow needed to capture CO_2 effectively from ambient air, generally fall between 1 and 6 m/s. This range is confirmed in Tegeler *et al.*,[33] where the mean velocity is reported as 3 m/s. Current DAC installations are estimated at 20 to 40 units, with new installations projected to increase by 10 to 30 units annually.[34] Utilising the total number of plants and the energy and specific fan power values discussed in Section S2.2.1, we estimate energy requirements for DAC systems to range from 400 to 800 W. This energy range accommodates variations in system size and efficiency, with specific fan power requirements between 1 and 2.5 W/(m^3/s) for effective operation.

S2.2.4 HVAC filters

The length of HVAC filter sheet is generally between 0.01 and 0.3 meters. In Schroth *et al.*,[35] for example, a HVAC filter sheet used in clean rooms is 0.28 meters long. We assume the number of pleats in a HVAC filter ranges from 100 to 300. According to Al *et al.*,[36] the number of pleats per 100 mm ranges from 28 to 34; with a filter width of 592 mm, this translates to 166 to 201 pleats, which aligns with our assumed range. This means that the surface area of the HVAC filter sheet (length $\times$ width $\times$ number of pleats) ranges from 0.2 to 200 m^2. Note that, e.g., when evaluating flow rates using the surface area, 0.2 m^2, the corresponding velocity must be used, 0.4 m/s, as the flow rate through the filter must remain constant, 0.08 m^3/s. HVAC filter efficiency is estimated between 95% and 100%, consistent with efficiencies reported for filter classes EU11 to EU14 in Schroth *et al.*[35] We estimate the total number of HVAC filters in use worldwide to be between 5×10^8 and 2×10^9, with annual production levels around 8×10^7 to 4×10^8. This estimation is consistent with the assumption that the number of filters aligns closely with the global building count, while the higher annual production reflects a growing demand for improved air quality. These estimates also align with data from Lowther *et al.*,[37] where the number of HVAC units sold in China in 2017 was 982 million, and the annual production was approximately 145 million.

S2.3 External flows

S2.3.1 Aeroplanes

The length of aeroplanes was estimated by considering a range of aircraft models, from the Boeing 737-700 to the Boeing 747-8, with lengths varying between approximately 20 m and

Table S4: The minimum, mean and maximum parameter values for length (m), surface area (m^2), velocity (m/s), total number (–), produced number (–), drag force (N) and drag coefficient (–). We consider flows around aeroplanes,[40–43] trains[44–47] and automobiles.[48–51]

Environment	Parameter	Ref.	Min.	Mean	Max.	Parameter	Ref.	Min.	Mean	Max.
Aeroplanes	Length (m)	40	20	40	80	Total number (−)	42	2×10^4	3×10^4	4×10^4
	Surface area (m^2)	40	150	300	1000	Produced number (−)	42	800	1×10^3	2×10^3
	Velocity (m/s)	41	200	250	300	Drag force (N)	43	1×10^4	5×10^4	2×10^5
	Drag coefficient (−)	43	0.02	0.03	0.05					
Trains	Length (m)	44	50	200	400	Total number (−)	46	1×10^5	2×10^5	3×10^5
	Surface area (m^2)	44	200	800	2×10^3	Produced number (−)	46	3×10^3	6×10^3	1×10^4
	Velocity (m/s)	45	40	60	100	Drag force (N)	47	2×10^3	1×10^4	3×10^4
	Drag coefficient (−)	47	0.2	0.3	0.4					
Automobiles	Length (m)	48	3.0	4.5	6.0	Total number (−)	50	1×10^9	2×10^9	3×10^9
	Surface area (m^2)	48	7	10	18	Produced number (−)	50	6×10^7	8×10^7	1×10^8
	Velocity (m/s)	49	30	35	50	Drag force (N)	51	1×10^2	3×10^2	1×10^3
	Drag coefficient (−)	51	0.2	0.4	0.7					

80 m. Values provided in McMasters *et al.*[40] (40–71 m) fall within this range. Using a typical fuselage diameter of 5 m, the estimated body surface area (area $\sim \pi \times$ radius $\times$ length) of aeroplanes ranges from $\pi \times 2.5 \times 20 = 1.6 \times 10^2$ m^2 to $\pi \times 2.5 \times 80 = 6 \times 10^2$ m^2, which we double to include the wing surface area. We assume the mean velocity to be between 200 m/s and 300 m/s. Subsonic civil/commercial aircraft examined in Filippone *et al.*[41] operate at Mach numbers ranging from 0.6 to 1, supporting these assumptions. The estimated total number of aeroplanes worldwide ranges between 2×10^4 and 4×10^4, with annual production between 5×10^2 and 2×10^3. These values are comparable to the number of flights per day in Europe, reported in Klower *et al.*[42] to vary between 2×10^4 and 4×10^4 over ten years. Drag forces for aircraft range from 1×10^4 N to 2×10^5 N, with drag coefficients between 0.01 and 0.05, reflecting the aerodynamic efficiency of various aircraft designs. In Sadraey *et al.*,[43] the drag force and drag coefficient range from 2×10^4 to 2×10^5 N and 0.02 to 0.04, respectively.

S2.3.2 Trains

Train lengths were estimated to range from 50 m for short urban light rail trains to 400 m for long-distance freight or high-speed trains. Assuming an average train car length of 20 m, the mode lengths in Schafer *et al.*,[44] ranging from 200 to 400 m, corroborate this estimate. With a typical train diameter of 4 m (double the standard gauge railway width of 2 m), the surface area is estimated to range from $4 \times 2 \times 50 = 4 \times 10^2$ m^2 to $4 \times 2 \times 400 = 3 \times 10^3$ m^2. Train speeds range from 40 m/s for commuter or freight trains to 100 m/s for high-speed rail systems. In Allotta *et al.*,[45] speeds are estimated to be between 40 and 70 m/s, consistent

with our assumptions. The total number of trains worldwide is estimated to be between 1×10^5 and 3×10^5, with annual production between 3×10^3 and 1×10^4. The number of trains in China is reported to be approximately 1×10^4, with annual production around 3×10^2, in line with global estimates if China is assumed to represent 10% of the world's trains.[46] We estimate the drag force for trains to range from 2×10^3 N to 3×10^4 N, with drag coefficients between 0.2 and 0.5, depending on the train design. In Mario *et al.*,[47] drag forces of 1×10^4 N and drag coefficients of 0.5 were reported, supporting our estimates.

S2.3.3 Automobiles

Automobile lengths were estimated to range from 3 m for smaller cars to 6 m for larger vehicles such as trucks. Values reported in Wasielewski *et al.*[48] for medium-length cars (4.6–5.5 m) fall within this range. The surface area was estimated using an average vehicle width of 2 m, with values ranging from 7 m^2 for smaller cars to 18 m^2 for larger vehicles with greater height. Velocity estimates for automobiles were based on typical highway speeds, with a minimum of 10 m/s and a maximum of 50 m/s. Wood *et al.*[49] provides velocity estimates between 30 and 50 m/s. The global number of automobiles is estimated to range between 1×10^9 and 3×10^9, with annual production between 6×10^7 and 1×10^8. Sperling *et al.*[50] estimates the global vehicle population approaching 2×10^9, with annual growth of about 3% (6×10^7). We assume that 10% of cars are in use at any given time, contributing to the estimated flow rates in our analysis. Drag forces for automobiles range from 100 N for small cars to 1000 N for large trucks, with drag coefficients between 0.2 and 0.4, depending on vehicle design. Sovran *et al.*[51] reports drag forces and coefficients between 68 and 774 N, and 0.3 to 0.6, respectively, aligning with these estimates.

S3 Methods

In this section, we provide a detailed explanation of the calculations used to estimate the flow rate of atmospheric pollutants to the surfaces of various applications, including cities, HVAC systems and transportation. These calculations are based on scaling theory (Section S3.1), empirical relationships (Section S3.2) and velocity and fully-developed profiles (Section S3.3). Additionally, we describe calculations that estimate the flow rate through the boundary layer, incorporating energy measurements, industry standards and drag measurements (Section S3.4).

S3.1 Scaling theory

This subsection presents a scaling theory for the pollutant flow rate to a flat surface, considering turbulent boundary layers. Consider a flat surface exposed to a fluid with a free-stream velocity u (m/s) and a characteristic length scale l (m). The total surface area of the flat surface is s (m^2), where n denotes the number of flat surfaces (–). Let c represent the pollutant concentration in the bulk fluid (mol/m^3) and D be the diffusion coefficient of the pollutant (m^2/s). The pollutant flux j_y (mol/(m^{2}s)) to the flat surface is given by the diffusive flux only, as the advective flux is zero, such that

$$j_y = -D \left(\frac{\partial c}{\partial y} \right)_{y=0}, \tag{1}$$

where $\partial c / \partial y$ is the concentration gradient normal to the surface at $y = 0$. Assuming a linear concentration profile in the boundary layer, the gradient at the surface can be approximated by

$$\left(\frac{\partial c}{\partial y} \right)_{y=0} \sim \frac{c}{\delta_c}. \tag{2}$$

Here, δ_c (m) denotes the pollutant boundary layer thickness. Therefore, the diffusive flux of the pollutant to the flat surface is

$$j_y \sim \frac{Dc}{\delta_c}. \tag{3}$$

The pollutant boundary layer thickness δ_c is related to the momentum boundary layer thickness δ through the Schmidt number Sc, defined as $Sc = \nu / D$, where ν is the kinematic viscosity of the fluid (m^2/s). The relationship is given by Frank *et al.*[52]

$$\delta_c \sim \frac{\delta}{Sc^{1/3}}. \tag{4}$$

The total pollutant flow rate q_y (mol/s) to the flat surface is obtained by multiplying the diffusive flux j_y by the surface area s

$$q_y = j_y ns \sim \frac{nsDc}{\delta_c}. \tag{5}$$

For turbulent flow, the turbulent diffusivity $D + D_e$ can be expressed in terms of the friction velocity, such that

$$D_e \sim u^* \delta. \tag{6}$$

Therefore, the pollutant flow rate to the flat surface is

$$q_y \sim \frac{ns(D+D_e)c}{\delta/Sc^{1/3}}. \tag{7}$$

Note that these approximations may not fully capture real-world complexities, particularly for PM, which is influenced by inertia and gravitational settling. These species may require different modelling approaches compared to GHGs.[53]

S3.2 Empirical relationships

This subsection presents empirical relationships for the boundary layer thickness, drag coefficient, Nusselt number and Sherwood number for a turbulent flow over a flat plate. All the empirical relations in this paragraph are taken from Frank *et al.*[52] The momentum boundary layer thickness is given by

$$\delta \approx \frac{0.4l}{Re^{1/5}}. \tag{8}$$

The Nusselt number Nu for heat transfer is

$$Nu \approx 0.03Re^{4/5}Pr^{1/3}. \tag{9}$$

The Sherwood number Sh for mass transfer is

$$Sh \approx 0.03Re^{4/5}Sc^{1/3}. \tag{10}$$

To relate heat and mass transfer, we use the relationship between the Nusselt and Sherwood numbers, along with the Prandtl and Schmidt numbers, defined as

$$\frac{Nu}{Pr^{1/3}} = \frac{Sh}{Sc^{1/3}}. \tag{11}$$

The mass transfer coefficient $m_c = ShD/l$ can be determined using

$$m_c \approx \frac{0.03Re^{4/5}Sc^{1/3}D}{l}, \tag{12}$$

using (10). Therefore, the total pollutant flow rate $q_y = j_y ns = m_c nsc$ to the flat surface can be calculated using

$$q_y \approx \frac{0.03 Re^{4/5} Sc^{1/3} nsDc}{l}. \tag{13}$$

It is important to note that empirical relationships may not fully capture the complexities of real-world systems, especially in cases involving irregular geometries, highly turbulent flows or non-standard flow regimes.[54] Under such conditions, deviations from theoretical predictions are expected and adjustments may be necessary to account for these influences.

S3.3 Fully-developed profiles

S3.3.1 Duct flow

For a fully-developed 2D channel flow centred around $y = 0$ with height $2H$, the velocity profile is described by $u(y)$. The pollutant flux to the channel wall is approximated by

$$j_y = -D\frac{\partial c}{\partial y}(0) \sim \frac{Dc}{h}. \tag{14}$$

Given that there are two walls and n channels, where s represents the surface area of each channel, the total pollutant flow rate to the channel walls can be expressed as

$$q_y \sim \frac{ns(D + D_e)c}{h}. \tag{15}$$

Note that in systems with bends or varying cross-sections, additional complexities arise, which can affect the accuracy of the predictions.

S3.3.2 HVAC filters

In this section, we derive the pollutant flow rates to the pleat walls and the fibre sheet in an HVAC filter, assuming the flow is fully developed. In Fig. 2G, the fibre sheet location varies linearly from the top to the bottom of the pleat. The velocity profile varies across the pleat channel and the velocity at the fibre sheet is given by $u_{\boldsymbol{n}}$. The velocities in the duct (u) and the filter sheet ($u_{\boldsymbol{n}}$) can be connected using conservation of mass

$$\int_A u \, \mathrm{d}A = \sum_{i=1}^{m} \int_{A_p} u_{\boldsymbol{n}} \, \mathrm{d}A_p, \tag{16}$$

where A is the cross-sectional area of the duct, A_p is the pleat area and there are m pleats in the HVAC filter. We match the average velocities to get

$$\bar{u}A \approx m\bar{u}_{\boldsymbol{n}}A_p. \tag{17}$$

The pollutant flow rate to the pleat walls is driven by diffusion, as discussed in Section S3.3.1. The rate of pollutant diffusion to the walls (area s_w, m^2) is expressed as

$$q_y \sim \frac{nms_w(D + D_e)c}{h}. \tag{18}$$

The pollutant flow rate through the fibre sheet, $q_{\boldsymbol{n}}$, is influenced by the collection efficiency E. The collection efficiency is commonly assumed to have an exponential dependence on quantities such as the local velocity near the fibre sheet, the collection parameter and the diffusivity of the fibre material.[55] For simplicity, we impose the values found in Tables S2–S4. The pollutant flow rate through the fibre sheet (area A_p, m^2) can be written as

$$q_{\boldsymbol{n}} \sim nmEA_p\bar{u}_{\boldsymbol{n}}c. \tag{19}$$

These formulas for pollutant flow rates to surfaces assume idealised conditions and do not account for real-world factors, such as airflow irregularities or particle variability, which may influence the actual performance of the systems.

S3.4 Power, industry standards and drag measurements

S3.4.1 Natural flows

This section describes the relationships between Q and q_y in the context of heat transfer, specifically focusing on the effects of heat flux and temperature difference, as dictated by energy conservation. We consider a flat surface with an imposed heat flux ϕ (W/m^2) over a specified area $s = lw$ (m^2). The total energy entering the control volume due to this surface heat flux can be expressed as

$$\text{Energy in} = \phi s. \tag{20}$$

This represents the energy input to the system per unit time, driving thermal processes in the surrounding environment. Next, we analyse the energy exiting the control volume. This energy outflow can be characterised by the mass flow rate $\dot{m}$ (kg/s), which is determined by the density ρ (kg/m^3), the free-stream velocity u (m/s) and the height of the control volume

δ (m)

$$\dot{m} = \rho u \delta w. \tag{21}$$

The energy leaving the control volume is then related to this mass flow rate and the specific heat capacity c_p (J/(kgK)) of the air, multiplied by the temperature difference ΔT (K) between the surface and the surrounding atmosphere. This relationship can be expressed as

$$\text{Energy out} = \dot{m} c_p \Delta T = (\rho u \delta w) c_p \Delta T. \tag{22}$$

According to the principle of conservation of energy, we can equate the energy entering the control volume, (20), to the energy exiting it, (21)–(22), such that

$$\phi s = \rho u \delta w c_p \Delta T. \tag{23}$$

Rearranging (23) to isolate $Q \sim u\delta w$, yields

$$Q = \frac{\phi s}{\rho c_p \Delta T}. \tag{24}$$

This expression provides a direct relationship between the streamwise flow rate and the physical parameters governing the heat transfer process. To derive an expression for the normal pollutant flow rate to the surface, q_y, we utilise the scaling theory described in (7). In order to relate q_y back to Q, we multiply and divide by Q to get

$$q_y \sim \frac{Qs(D + D_e)c}{\delta^2 uw}. \tag{25}$$

Fig. S1 presents the variation in wind velocity and sensible heat flux across the world. Data for the wind velocity, boundary layer thickness, sensible heat flux and temperature is collected from ERA5,[56] with the world's cities and solar farms superimposed on top using SimpleMaps[57] and Dunnett *et al.*[58] respectively. Equation (24) provides a useful method for estimating airflow rates in scenarios influenced by thermal gradients, such as urban heat islands or solar energy collection. However, this approach may fail in highly turbulent conditions where the assumptions of steady-state flow do not hold.[59]

S3.4.2 Internal flows

Duct and filter leakage can affect the performance and energy efficiency of HVAC systems and HVAC filters.[60] Typical duct leakage in HVAC systems ranges from 5% to 20%, depending

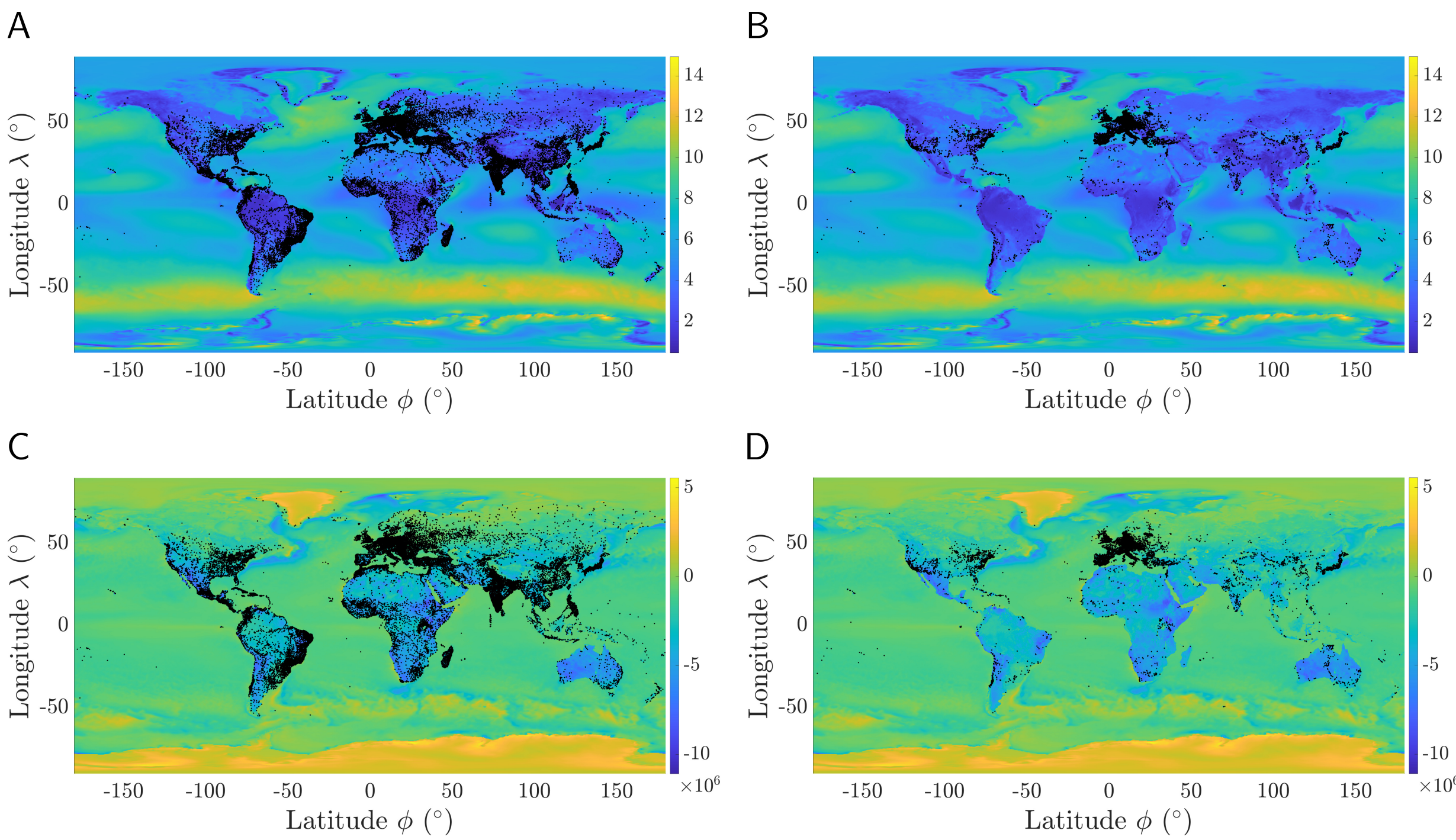

Figure S1: Contours of the global wind speed (m/s) distribution with the (A) world's cities in black and (B) world's solar farms in black, along with contours of the global sensible heat flux (W/m^2) distribution with the (C) world's cities in black and (D) world's solar farms in black.[56–58]

on factors such as installation quality and system maintenance, with 10% being a common assumption for well-maintained systems. For HVAC filters, when properly installed, leakage is typically negligible and below 1%. However, poorly installed systems can experience leakage rates as high as 3%. To account for these losses in flow rate and energy calculations, a leakage factor should be applied. This factor reduces the estimated airflow and adjusts energy consumption based on the system's specific leakage rate. Incorporating these leakage factors ensures more accurate estimates of system performance and energy use. Note that these methods may be less accurate in situations with significant leakage or fluctuations in occupancy, complex building geometries or varying external conditions.

S3.4.3 External flows

For external flows over transportation systems, the streamwise flow rate Q can be estimated through the application of drag force dynamics.[61] The drag force F_d (N) experienced by an object moving through a fluid is influenced by both the properties of the fluid and the object itself,

$$F_d = \frac{1}{2} C_d \rho u^2 A, \tag{26}$$

where C_d is the drag coefficient (–), ρ is the fluid density (kg/m^3), u is the average velocity of the object relative to the fluid (m/s) and A is the reference area (m^2) of the object exposed to the flow. To derive Q in terms of F_d, we rearrange (26) to incorporate $Q = Au$, yielding

$$F_d = \frac{C_d Q \rho u}{2}. \tag{27}$$

From this, the streamwise flow rate can be expressed as a function of the drag force acting on the transportation system

$$Q = \frac{2F_d}{\rho u C_d}. \tag{28}$$

The normal pollutant flow rate to the surface of the external flow can be evaluated using (25). Note that the above flow-rate formulas may fail to provide accurate estimates in scenarios involving complex flow patterns, such as turbulence, vortex shedding or crosswinds.

S4 The feasibility of different applications for surface-based atmospheric pollutant removal

S4.1 Solar panels

Coating the surfaces of solar panels with sorbent or photocatalytic materials may reduce electrical output by partially occluding the panel surface. However, transparent TiO_2 coatings on solar panels have been demonstrated for NO_x removal while largely preserving optical transmission,[62] although their pollutant removal performance may differ from that reported for street and wall applications.[63] Solar farms are included primarily to illustrate potential atmospheric pollutant flow to large engineered surfaces, rather than as a practical strategy for atmospheric pollutant removal.

S4.2 HVAC systems

HVAC systems are used here as representative high-airflow environments to estimate transport-limited pollutant delivery rates to surfaces. In ventilation environments, low-temperature technologies such as sorbents or photocatalysts would be more compatible with existing HVAC operation than high-temperature thermocatalysts. Photocatalytic systems require photon flux to drive oxidation reactions. UV irradiation is already deployed in some HVAC systems for microbial control, with characteristic doses of approximately 0.1 to 496 J/m^2

over typical exposure times of 0.1 to 16 s,[64] corresponding to irradiance levels of approximately 0.4 to 112 W/m^2. This range overlaps with the irradiance levels of 10 to 100 W/m^2 reported in photocatalyst studies for CH_4 oxidation.[65] However, practical implementation would depend on wavelength compatibility, residence time, catalyst efficiency and the energy cost of UV illumination, none of which are captured in the transport-limited estimates presented here.

S4.3 HVAC filters

HVAC filters progressively accumulate PM during operation, forming a filter cake that increases pressure drop and necessitates periodic replacement. Typical HVAC filters are replaced every 1 to 3 months depending on building type, outdoor air quality and operating conditions, with increasing PM loading raising fan energy demand prior to replacement. This periodic replacement is already standard practice in HVAC operation and could in principle provide an existing pathway for deploying sorbents or catalysts without introducing additional maintenance cycles; it may also help maintain catalytic or sorbent activity over time relative to static outdoor deployments. If sorbents or catalysts are incorporated into HVAC filters, accumulated PM may block active sites and reduce removal efficiency.[66] Sorbent saturation or catalyst deactivation may also occur independently of fouling, so material lifetime is governed by chemical and physical degradation mechanisms. For example, under quarterly replacement, \$600 per tCO_2 for a single installation cycle would increase to \$2400 per tCO_2 removed, with further increases for more frequent replacement or declining performance between cycles. Quantifying the coupled effects of filtration, fouling and material degradation is beyond the scope of this study but remains an important direction for future work.

S4.4 Aeroplane wings and turbine blades

Surfaces such as wind turbine blades and aircraft wings are subject to strict aerodynamic and structural constraints; surface properties and mass distribution are critical to performance and lifetime, and any coating would need to satisfy these requirements. Wind turbine blades and aircraft wings are included primarily to illustrate atmospheric flow to surfaces, rather than to suggest practical pollutant removal at these surfaces.

S5 O_3 using catalysis

Although our main analysis focused on CO_2, CH_4, $PM_{2.5}$ and NO_x, the methodology is general and can be applied to many atmospheric pollutants and removal technologies. One

must specify the pollutant's atmospheric concentration, diffusivity, surface area and airflow of the environment, calculating the atmospheric flow rate using Methods and applying the removal efficiency of the chosen technology. For example, O_3 is a short-lived climate pollutant linked to respiratory illness and crop yield losses,[67] with typical atmospheric concentrations of 60 ppb (3×10^{-6} mol/m^3) and diffusivity of 2×10^{-5} m^2/s. Using these values in (13) for city environments (Tab. S2), gives an atmospheric flow rate to all city surfaces of around 3×10^3 mol/s. With a photocatalytic removal efficiency of 0.5 given by Ohtani et al.[68] for TiO_2, the above flow rate corresponds to a removal potential of 2.3×10^{-3} GtO_3/y, representing approximately 1% of the tropospheric O_3 burden of around 0.4 Gt.[67]

A relevant real-world example of catalytic pollutant removal is the PremAir™ radiator coating deployed in certain vehicles in the 1990s.[69] This system incorporated a catalyst into the vehicle radiator to decompose ambient O_3 as air passed through the cooling airflow. Laboratory experiments reported O_3 removal efficiencies of up to 90%. However, fleet-scale analyses concluded that the resulting reduction in urban O_3 concentrations was modest because the catalytic surface area and airflow rate represented only a small fraction of the atmospheric volume, and deployment was difficult to justify without strong regulatory incentives.

S6 Produced flow rate

In Fig. S2, we present the flow rate of atmospheric CO_2, CH_4, $PM_{2.5}$ and NO_x to the produced surfaces of cities, solar farms, HVAC systems, combustion systems, DAC systems, aeroplanes, trains and automobiles. This figure can be compared with the flow rate of atmospheric pollutants to the total surfaces in Fig. 3 of the main text.

S7 Other climate metrics

Finally, we note that the 100-year GWP remains a commonly used regulatory and corporate reporting metric. Under the 100-year GWP, methane's CO_2-equivalent factor is approximately 27 to 30, compared with approximately 80 to 84 under the 20-year GWP.[70] Consequently, the CO_2e benefit per tonne of CH_4 potentially removed would be approximately three times lower than the values reported here, and the cost per tCO_2e potentially removed would increase proportionally. Alternative metrics may better represent specific policy or temperature-based accounting objectives. While the choice of equivalency metric affects the interpretation of CO_2e benefits, it does not alter the atmospheric pollutant fluxes to surfaces estimated in this study.

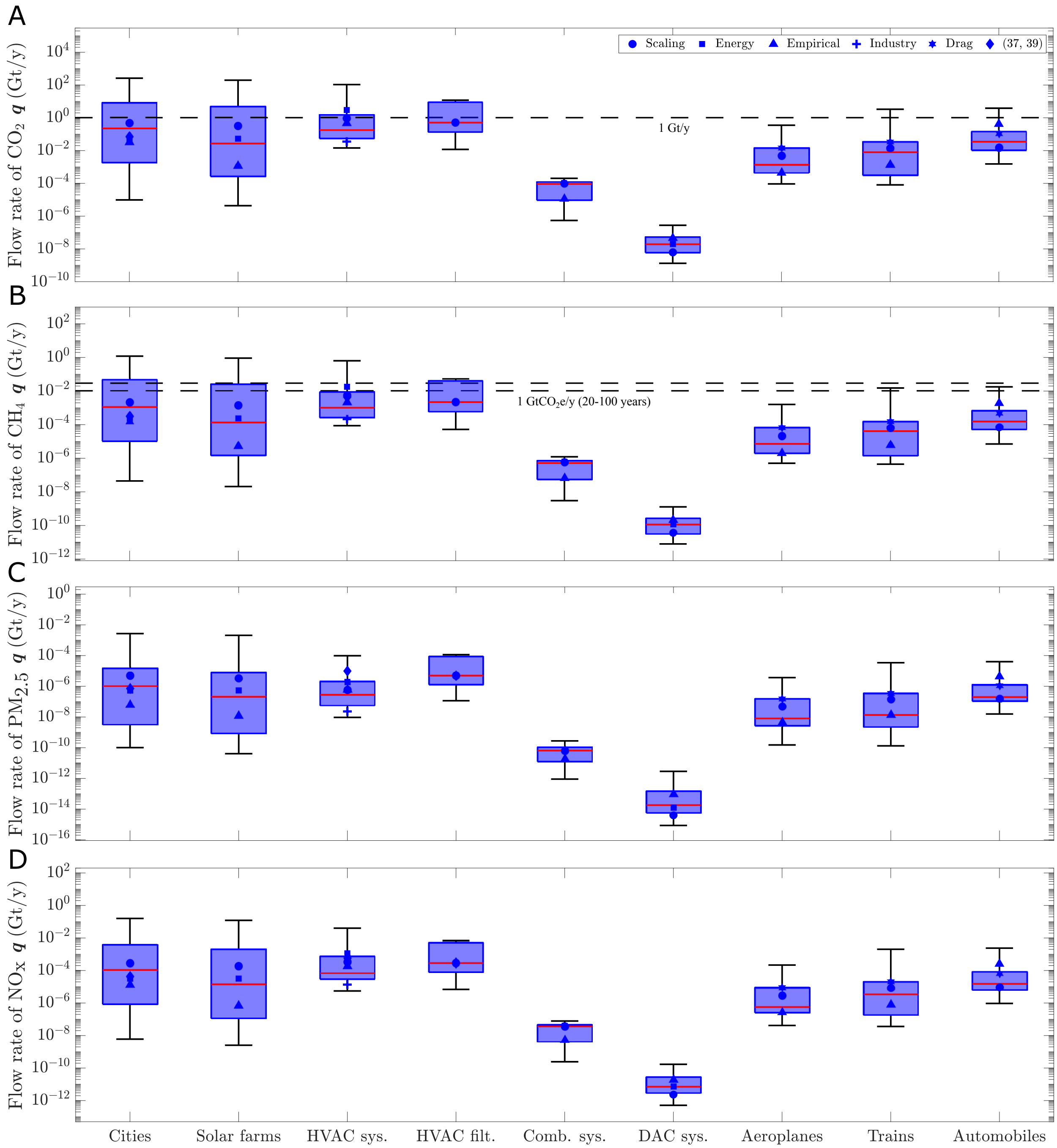

Figure S2: Flow rates ($\boldsymbol{q}$) of atmospheric (A) CO_2, (B) CH_4, (C) $PM_{2.5}$ and (D) NO_x to the annually produced surfaces in natural (e.g., cities, solar farms), internal (e.g., HVAC, combustion, DAC systems) and external (e.g., aeroplanes, trains, automobiles) environments, with symbols representing the methods used to estimate flow rates.

References

(1) Britter, R. E.; Hanna, S. R. Flow and dispersion in urban areas. *Annu. Rev. Fluid Mech.* **2003**, *35*, 469–496.

(2) Bettencourt, L. M. A.; Lobo, J. Urban scaling in Europe. *J. R. Soc. Interface* **2016**, *13*, 20160005.

(3) Holzworth, G. C. *Mixing heights, wind speeds, and potential for urban air pollution throughout the contiguous United States*; US Government Printing Office, 1972.

(4) Angel, S.; Parent, J.; Civco, D. L.; Blei, A.; Potere, D. The dimensions of global urban expansion: Estimates and projections for all countries, 2000–2050. *Prog. Plann.* **2011**, *75*, 53–107.

(5) Allen, L.; Lindberg, F.; Grimmond, C. S. B. Global to city scale urban anthropogenic heat flux: model and variability. *Int. J. Climatol.* **2011**, *31*, 1990.

(6) Erell, E.; Williamson, T. Intra-urban differences in canopy layer air temperature at a mid-latitude city. *Int. J. Climatol.* **2007**, *27*, 1243–1255.

(7) Giardina, M.; Donateo, A.; Buffa, P.; Contini, D.; Cervone, A.; Lombardo, C.; Rocchi, F. Atmospheric dry deposition processes of particles on urban and suburban surfaces: Modelling and validation works. *Atmos. Environ.* **2019**, *214*, 116857.

(8) Friedlingstein, P.; O'Sullivan, M.; Jones, M. W.; Andrew, R. M.; Gregor, L.; Hauck, J.; Le Quéré, C.; Luijkx, I. T.; Olsen, A.; Peters, G. P.; others Global carbon budget 2022. *Earth Syst. Sci. Data* **2022**, *14*, 4811–4900.

(9) Dlugokencky, E. J.; Nisbet, E. G.; Fisher, R.; Lowry, D. Global atmospheric methane: budget, changes and dangers. *Philos. Trans. R. Soc. A* **2011**, *369*, 2058–2072.

(10) Tian, H.; Pan, N.; Thompson, R. L.; Canadell, J. G.; Suntharalingam, P.; Regnier, P.; Davidson, E. A.; Prather, M.; Ciais, P.; Muntean, M.; others Global nitrous oxide budget 1980–2020. *Earth Syst. Sci. Data Disc.* **2023**, *2023*, 1–98.

(11) Organization, W. H.; others *WHO global air quality guidelines: particulate matter (PM2.5 and PM10), ozone, nitrogen dioxide, sulfur dioxide and carbon monoxide*; World Health Organization, 2021.

(12) Manisalidis, I.; Stavropoulou, E.; Stavropoulos, A.; Bezirtzoglou, E. Environmental and health impacts of air pollution: a review. *Front. Public Health* **2020**, *8*, 14.

(13) Boningari, T.; Smirniotis, P. G. Impact of nitrogen oxides on the environment and human health: Mn-based materials for the NO_x abatement. *Curr. Opin. Chem. Eng.* **2016**, *13*, 133–141.

(14) Steadman, P.; Evans, S.; Batty, M. Wall area, volume and plan depth in the building stock. *Build. Res. Inf.* **2009**, *37*, 455–467.

(15) Davies, R. G.; Barbosa, O.; Fuller, R. A.; Tratalos, J.; Burke, N.; Lewis, D.; Warren, P. H.; Gaston, K. J. City-wide relationships between green spaces, urban land use and topography. *Urban Ecosyst.* **2008**, *11*, 269–287.

(16) Woolway, R. I.; Zhao, G.; Rocha, S. M. G.; Thackeray, S. J.; Armstrong, A. Decarbonization potential of floating solar photovoltaics on lakes worldwide. *Nat. Water* **2024**, *2*, 566–576.

(17) Jones, P.; Hillier, D.; Comfort, D. Solar farm development in the United Kingdom. *Prop. Manag.* **2014**, *32*, 176–184.

(18) Hossain, M. D.; Wadi Al-Fatlawi, A.; Kumar, L.; Fang, Y. R.; Assad, M. E. H. Solar PV high-penetration scenario: an overview of the global PV power status and future growth. **2024**, *15*, 1–57.

(19) Glick, A.; Smith, S. E.; Ali, N.; Bossuyt, J.; Recktenwald, G.; Calaf, M.; Cal, R. B. Influence of flow direction and turbulence intensity on heat transfer of utility-scale photovoltaic solar farms. *Sol. Energy* **2020**, *207*, 173–182.

(20) Yang, L.; Gao, X.; Lv, F.; Hui, X.; Ma, L.; Hou, X. Study on the local climatic effects of large photovoltaic solar farms in desert areas. *Solar Energy* **2017**, *144*, 244–253.

(21) Saad, N. M.; Shukair, M. K. A. M.; Saraf, N. M.; Hamid, J. R. A.; Talib, N.; Rasam, A. R. A. The potential area estimation for solar installation based on insolation pattern. Charting the Sustainable Future of ASEAN in Science and Technology: Proceedings from the 3rd International Conference on the Future of ASEAN (ICoFA) 2019-Volume 2. 2020; p 335.

(22) Fisk, W. J.; Delp, W. W.; Diamond, R.; Dickerhoff, D.; Levinson, R.; Modera, M.; Nematollahi, M.; Wang, D. Duct systems in large commercial buildings: physical characterization, air leakage, and heat conduction gains. *Energy Build.* **2000**, *32*, 109–119.

(23) Fisk, W. J.; Faulkner, D.; Sullivan, D. P. *An evaluation of technologies for real-time measurement of rates of outdoor airflow into HVAC systems*; 2004.

(24) Knight, I. P. Assessing electrical energy use in HVAC systems. *REHVA J.* **2012**, *49*, 6–11.

(25) Merzkirch, A.; Maas, S.; Scholzen, F.; Waldmann, D. Field tests of centralized and decentralized ventilation units in residential buildings: Specific fan power, heat recovery efficiency, shortcuts and volume flow unbalances. *Energy Build.* **2016**, *116*, 376–383.

(26) Eto, J. H. The HVAC Costs of Increased Fresh Air Ventilation Rates in Office Buildings, Part 2. Proc. 5th Int. Conf. Indoor Air Quality and Climate. 1990.

(27) Herrmann, M. The global population will soon reach 8 billion—then what. *UN Chronicle* **2022**, *59*, Available online: https://www.un.org/en/un-chronicle/global-population-will-soon-reach-8-billion-then-what.

(28) Persily, A. K.; Gorfain, J. *Analysis of ventilation data from the US Environmental Protection Agency building assessment survey and evaluation (BASE) study*; U.S. Department of Commerce, Technology Administration, National Institute of Standards and Technology, 2004.

(29) Memarzadeh, F.; Xu, W. Role of air changes per hour (ACH) in possible transmission of airborne infections. Building Simulation. 2012; pp 15–28.

(30) Hale, M. E.; Brittain, F. Effect of alternative combinations of source type, sizes, and complexity on accuracy of modeling a power plant. INTER-NOISE and NOISE-CON Congress and Conference Proceedings. 2014; pp 679–688.

(31) Zhang, H.; Yue, G.; Lu, J.; Jia, Z.; Mao, J.; Fujimori, T.; Suko, T.; Kiga, T. Development of high temperature air combustion technology in pulverized fossil fuel fired boilers. *Proc. Combust. Inst.* **2007**, *31*, 2779–2785.

(32) Brief, C. Mapped: The world's coal power plants in 2020. 2020; https://www.carbonbrief.org/mapped-worlds-coal-power-plants/, Accessed: October 18, 2024.

(33) Tegeler, E.; Cui, Y.; Masoudi, M.; Bahmanpour, A. M.; Colbert, T.; Hensel, J.; Balakotaiah, V. A novel contactor for reducing the cost of direct air capture of CO2. *Chem. Eng. Sci.* **2023**, *281*, 119107.

(34) International Energy Agency Direct Air Capture. 2023; https://www.iea.org/energy-system/carbon-capture-utilisation-and-storage/direct-air-capture, Accessed: 2024-10-18.

(35) Schroth, T. New HEPA/ULPA filters for clean-room technology. *Filtr. Separ.* **1996**, *33*, 245–244.

(36) Al-Attar, I. S.; Wakeman, R.; Tarleton, E.; Husain, A. The effect of pleat count and air velocity on the initial pressure drop and fractional efficiency of HEPA filters. *In: Proc. Filtech* **2009**, *9*, 19–26.

(37) Lowther, S. D.; Deng, W.; Fang, Z.; Booker, D.; Whyatt, J. D.; Wild, O.; Wang, X.; Jones, K. C. Factors affecting real-world applications of HEPA purifiers in improving indoor air quality. *Environ. Sci. Adv.* **2023**, *2*, 235–246.

(38) McQueen, N.; Gomes, K. V.; McCormick, C.; Blumanthal, K.; Pisciotta, M.; Wilcox, J. A review of direct air capture (DAC): scaling up commercial technologies and innovating for the future. *Prog. Energy* **2021**, *3*, 032001.

(39) Sippola, M. R.; Nazaroff, W. W. *Particle deposition from turbulent flow: Review of published research and its applicability to ventilation ducts in commercial buildings*; 2002.

(40) McMasters, J. H.; Kroo, I. M. Advanced configurations for very large transport airplanes. *Aircraft Des.* **1998**, *1*, 217–242.

(41) Filippone, A. Data and performances of selected aircraft and rotorcraft. *Prog. Aerosp. Sci.* **2000**, *36*, 629–654.

(42) Klöwer, M.; Allen, M. R.; Lee, D. S.; Proud, S. R.; Gallagher, L.; Skowron, A. Quantifying aviation's contribution to global warming. *Environ. Res. Lett.* **2021**, *16*, 104027.

(43) Sadraey, M. H. *Aircraft Performance Analysis*; VDM Verlag Dr. Müller, 2009; pp 123–145.

(44) Schafer, D. H.; Barkan, C. P. L. Relationship between train length and accident causes and rates. *Transp. Res. Rec.* **2008**, *2043*, 73–82.

(45) Allotta, B.; Colla, V.; Malvezzi, M. Train position and speed estimation using wheel velocity measurements. *Proc. Inst. Mech. Eng. F* **2002**, *216*, 207–225.

(46) Singh, S. *New Mega Trends: Implications for Our Future Lives*; Springer, 2012; pp 181–200.

(47) Mario, J.; Halfina, B.; Bahtera, D.; Shalahuddin, L.; Windharto, A. Numerical and experimental analysis of drag force in medium-speed train design. IOP Conf. Ser.: Mater. Sci. Eng. 2020; p 012031.

(48) Wasielewski, P. The effect of car size on headways in freely flowing freeway traffic. *Transp. Sci.* **1981**, *15*, 364–378.

(49) Wood, D. P.; Ydenius, A.; Adamson, D. Velocity changes, mean accelerations and displacements of some car types in frontal collisions. *Int. J. Crashworthiness* **2003**, *8*, 591–603.

(50) Sperling, D.; Gordon, D. *Two Billion Cars: Driving Toward Sustainability*; Oxford University Press, 2009.

(51) Sovran, G. *Aerodynamic Drag Mechanisms of Bluff Bodies and Road Vehicles*; Springer Science & Business Media, 2012.

(52) Frank, P. Incropera's Principles of Heat and Mass Transfer. 2017.

(53) Turpin, B. J.; Saxena, P.; Andrews, E. Measuring and simulating particulate organics in the atmosphere: problems and prospects. *Atmos. Environ.* **2000**, *34*, 2983–3013.

(54) Schlichting, H.; Gersten, K. *Boundary-layer theory*; Springer, 2016.

(55) Hinds, W. C.; Zhu, Y. *Aerosol Technology: Properties, Behavior, and Measurement of Airborne Particles*; John Wiley & Sons, 2022.

(56) Hersbach, H.; Bell, B.; Berrisford, P.; Hirahara, S.; Horányi, A.; Muñoz-Sabater, J.; Nicolas, J.; Peubey, C.; Radu, R.; Schepers, D.; others ERA5 reanalysis (single levels). 2017; Copernicus Climate Change Service (C3S) Data Store (CDS), accessed 2024. https://cds.climate.copernicus.eu/datasets/reanalysis-era5-single-levels.

(57) SimpleMaps World Cities Database. 2024; https://simplemaps.com/data/world-cities, Accessed: 2024-10-17.

(58) Dunnett, S.; Sorichetta, A.; Taylor, G.; Eigenbrod, F. Harmonised global datasets of wind and solar farm locations and power. *Sci. Data* **2020**, *7*, 130.

(59) Oke, T. R. *Boundary Layer Climates*; Routledge, 2002.

(60) Schild, P. G.; Mysen, M. Recommendations on specific fan power and fan system efficiency. *Tech. Note AIVC* **2009**, *65*.

(61) White, F. M.; Xue, H. *Fluid mechanics*; McGraw-Hill, 2003; Vol. 3.

(62) Molar, J.; Herckes, P.; Fraser, M. P. Photocatalytic abatement of ambient NOx by TiO2 coated solar panels. *RSC Sustain.* **2025**, *3*, 963–972.

(63) Russell, H. S.; Frederickson, L. B.; Hertel, O.; Ellermann, T.; Jensen, S. S. A review of photocatalytic materials for urban NOx remediation. *Catalysts* **2021**, *11*, 675.

(64) Thornton, G. M.; Fleck, B. A.; Fleck, N.; Kroeker, E.; Dandnayak, D.; Zhong, L.; Hartling, L. The impact of heating, ventilation, and air conditioning design features on the transmission of viruses, including the 2019 novel coronavirus: A systematic review of ultraviolet radiation. *PLoS One* **2022**, *17*, e0266487.

(65) Tsopelakou, A. M.; Stallard, J.; Archibald, A. T.; Fitzgerald, S.; Boies, A. M. Exploring the bounds of methane catalysis in the context of atmospheric methane removal. *Environ. Res. Lett.* **2024**, *19*, 054020.

(66) Tomlinson, S. D.; Tsopelakou, A. M.; Onn, T. M.; Barrett, S. R. H.; Boies, A. M.; Fitzgerald, S. Modelling laminar flow in V-shaped filters integrated with catalyst technologies for atmospheric pollutant removal. *Int. J. Heat Mass Transf.* **2026**, *257*, 128206.

(67) Zhang, J.; Wei, Y.; Fang, Z. Ozone pollution: a major health hazard worldwide. *Front. Immunol.* **2019**, *10*, 2518.

(68) Ohtani, B.; Zhang, S. W.; Nishimoto, S.; Kagiya, T. Catalytic and photocatalytic decomposition of ozone at room temperature over titanium (IV) oxide. *J. Chem. Soc., Faraday Trans.* **1992**, *88*, 1049–1053.

(69) Greger, L.; Bergqvist, M.; Gottberg, I.; Wirmark, G.; Heck, R.; Hoke, J.; Anderson, D.; Rudy, W.; Adomaitis, J. *PremAir® Catalyst System*; 1998.

(70) Balcombe, P.; Speirs, J. F.; Brandon, N. P.; Hawkes, A. D. Methane emissions: choosing the right climate metric and time horizon. *Environ. Sci. Process. Impacts* **2018**, *20*, 1323–1339.
